# ﻿Multi-omics insights into growth and fruiting body development in the entomopathogenic fungus *Cordycepsblackwelliae*

**DOI:** 10.3897/imafungus.16.147558

**Published:** 2025-05-07

**Authors:** Jia-Ni Li, Shu Zhang, Yong-Jie Zhang

**Affiliations:** 1 School of Life Science, Shanxi University, Taiyuan 030006, China Shanxi University Taiyuan China; 2 Key Laboratory of Microbial Resources Collection and Preservation, Ministry of Agriculture and Rural Affairs, Beijing 100081, China School of Life Science, Shanxi University, Taiyuan, 030006, China Taiyuan China; 3 Key Laboratory of Chemical Biology and Molecular Engineering of Ministry of Education, Shanxi University, Taiyuan 030006, China Key Laboratory of Microbial Resources Collection and Preservation, Ministry of Agriculture and Rural Affairs Beijing China

**Keywords:** *Cordycepsblackwelliae*, fruiting body development, genome, transcriptome, metabolome

## Abstract

*Cordycepsblackwelliae* is an entomopathogenic fungus with significant potential for research and development due to its ease of cultivation. However, the lack of omics-based studies has limited our understanding of the molecular mechanisms governing its growth and fruiting body development. This study employed a multi-omics approach, integrating genomic, transcriptomic and metabolomic analyses. Utilising both Illumina and Nanopore sequencing technologies, we assembled a 31.06 Mb nuclear genome comprising 11 scaffolds, with telomere presence at one or both ends in eight scaffolds and annotated 8,138 identified genes (8,136 from genome prediction and two from local BLAST searches). Transcriptomic analysis identified 2,078 differentially expressed genes across three developmental stages: liquid culture mycelia, wheat culture mycelia and fruiting bodies. Amongst these, 745 genes were up-regulated in fruiting bodies, primarily associated with biosynthetic and catabolic pathways. Metabolomic analysis identified 1,161 metabolites, with 1,014 showing significant variations across developmental stages. Integrated transcriptomic and metabolomic analyses uncovered 17 genes positively correlated with 34 metabolites, which are likely crucial regulators of fruiting body development. These findings provide new insights into the molecular networks underlying *C.blackwelliae* growth and fruiting body formation.

## ﻿Introduction

The genus *Cordyceps* comprises approximately 180 recognised species of entomopathogenic fungi that infect insects (Zhang et al. 2023). Due to overharvesting, wild *Cordyceps* resources have become increasingly scarce (Wei et al. 2021). To overcome this limitation, advanced artificial cultivation techniques have been established for large-scale production of mycelia, fruiting bodies and bioactive metabolites (Li et al. 2019). Currently, the mycelia and fruiting bodies of *Cordycepschanhua* and *Cordycepsmilitaris* have been widely exploited for food, health products or medicines (Dong et al. 2015; Chen et al. 2020). To meet the growing demand, more *Cordyceps* species need to be explored and developed.

*Cordycepsblackwelliae* is an entomopathogenic fungus first described in 2018 (Mongkolsamrit et al. 2018) and subsequently documented in China, Thailand and Vietnam (Duan 2019). Fruiting bodies of *C.blackwelliae* can be readily cultivated on artificial substrates and insect pupae and they exhibit antioxidant, antibacterial and fibrinolytic properties (Fan et al. 2022). Although its mitochondrial genome has been characterised (Zhang et al. 2023), the nuclear genome of *C.blackwelliae* remains unidentified. Due to its ease of cultivation and potential applications, *C.blackwelliae* presents a promising model organism for investigating the mechanisms underlying fungal growth and fruiting body development.

Throughout its cultivation cycle, *C.blackwelliae* undergoes distinct morphological and physiological adaptations across three developmental stages. During the liquid culture mycelium (LM) phase, the fungus forms dense, filamentous biomass characterised by rapid growth and high antioxidant capacity, making it ideal for industrial fermentation and metabolic investigations (Dong et al. 2015; Li et al. 2025a). In the wheat culture mycelium (WM) phase, the fungus develops complex structural networks that closely mimic natural growth conditions. Finally, in the fruiting body (FB) stage, the fungus undergoes morphological differentiation to form specialised reproductive structures while accumulating bioactive compounds (Zheng et al. 2011). Elucidating the molecular mechanisms driving these developmental transitions is crucial for unravelling the regulatory networks underlying fungal morphogenesis in *C.blackwelliae*.

A multi-omics strategy integrating genomic, transcriptomic and metabolomic analyses provides a comprehensive framework for systematically investigating the molecular mechanisms underlying fungal growth and fruiting body development. Genomics enables the construction of high-quality reference genomes, facilitating the identification of key genes and regulatory elements involved in fungal growth and development (Shen et al. 2024). While next-generation sequencing technologies have been widely used for several *Cordyceps* genome projects (Suppl. material 1: table S1), they often produce fragmented genome assemblies that may exclude critical genetic elements (Chen et al. 2017). To overcome this limitation, hybrid sequencing approaches that integrate both next-generation and third-generation sequencing technologies have been employed (Lei et al. 2023). This strategy has been successfully applied to *C.militaris* CMS19, resulting in a highly contiguous genome assembly (Chai et al. 2024). Transcriptomic analyses provide insights into the molecular mechanisms underlying fungal growth and fruiting body development. For example, comparative transcriptomic analyses of *Ophiocordycepssinensis* at various developmental stages have revealed key insights into the energy supply mechanisms required for fruiting body development (He et al. 2023). Similarly, transcriptomic profiling of *C.militaris* mycelia and fruiting bodies has highlighted the importance of signal transduction and metabolic pathways in fruiting body formation (Yin et al. 2012). Metabolomics further enhances our understanding of fungal development by identifying key metabolites associated with reproductive growth and spore maturation. For instance, studies on *Cordycepscicadae* have identified nicotinamide and gibberellin as crucial metabolites for reproductive development (Qu et al. 2019). However, existing research on *Cordyceps* metabolites primarily focuses on their nutritional and medicinal properties across different species (Liu et al. 2021; Wang et al. 2023), leaving a substantial knowledge gap in understanding the metabolic regulation of fruiting body formation. Integrating metabolomic and transcriptomic approaches has proven to be a powerful strategy for elucidating molecular networks governing fungal development and maturation in *Ophiocordycepssinensis* (He et al. 2023) and *Dictyophoraindusiata* (Duan et al. 2023). Applying these methodologies to *C.blackwelliae* could significantly enhance our understanding of fruiting body differentiation.

Fungal growth and development are governed by complex, multi-level regulatory systems. We hypothesise that the growth and fruiting body development of *C.blackwelliae* are modulated at both the transcriptional and metabolic levels. To test this hypothesis, we performed whole genome sequencing of *C.blackwelliae* and conducted multi-omics analyses (transcriptomics and metabolomics) across three key developmental stages: LM, WM and FB. Specifically, this study aims to: 1) assemble and annotate the first nuclear genome of *C.blackwelliae*; 2) investigate transcriptional patterns across different developmental stages; 3) identify key metabolites associated with fungal growth and fruiting body formation; and 4) explore the relationship between differentially expressed genes (DEGs) and differential metabolites (DMs). This research provides a foundational understanding of the intrinsic molecular mechanisms regulating *C.blackwelliae* growth and fruiting body development, paving the way for future studies on fungal morphogenesis and bioactive compound production.

## ﻿Materials and methods

### ﻿Fungal strain and sample preparation

The *Cordycepsblackwelliae* strain ZYJ0835 used in this study was isolated from a fresh specimen collected from Guangdong, China in 2019 and deposited at the China Center for Type Culture Collection (CCTCC) under no. M2022663 (Fan et al. 2022). The strain was grown on potato dextrose agar (PDA) overlaid with sterile cellophane at 25°C for seven days. Mycelia were harvested for genomic DNA extraction using the cetyl trimethyl ammonium bromide method (Zhang et al. 2010). To prepare samples for RNA sequencing and metabolic analysis, fungal materials were prepared at three distinct growth stages in accordance with the procedures described in our previous study (Fan et al. 2022). Briefly, the fungus was cultivated at 25°C and 160 r/min for six days using the potato dextrose broth medium to obtain the liquid culture mycelia (LM), which represents the undifferentiated, fast-growing vegetative phase. The wheat culture mycelia (WM) were obtained by inoculating the fungus to wheat media in glass bottles and cultivating the fungus under dark conditions at 20°C with humidity levels of 60–70%. Mycelia were collected when the mycelia reached full growth across the wheat substrate. This stage mimics the natural substrate colonisation phase where invasive growth initiation occurs. To induce fruiting body (FB) formation, duplicate WM cultures were exposed to 200 lux fluorescent light with alternating temperature (22°C for 14 h and 17°C for 10 h) to stimulate primordial formation. After primordia differentiated, the fungus was kept at 22°C with 80% relative humidity and received a daily photoperiod of 12 hours under a light intensity of 1,000 lux. Fruiting bodies were then ready to be harvested when they grew to 3–5 cm long. The FB stage represents the reproductive phase, crucial for studying developmental morphogenesis. All collected samples were promptly frozen in liquid nitrogen and stored at -80°C until further use. Total RNA was extracted using Trizol reagent (DP424, Tiangen Biotech Co., Ltd., Beijing, China) with quantity verification performed using an Agilent 2100 bioanalyser (Agilent Technologies, Palo Alto, CA, USA).

### ﻿Genome assembly and annotation

Genome sequencing was performed by Majorbio Bio-Pharm Technology Co., Ltd. (Shanghai, China) using both Illumina NovaSeq 6000 (PE150) and Nanopore PromethION R9.4 platforms. Illumina data underwent quality control filtering by fastp v.0.23.0 (Chen et al. 2018) to remove low-quality reads. For Nanopore data, quality control was conducted using Guppy v.4.3.4 within the MinKNOW v.2.2 package for base calling and only reads with an average Q score exceeding seven were selected for subsequent analyses. Genome assembly was performed firstly from the Nanopore data using CANU assembler v.1.7 (Koren et al. 2017), followed by polishing with the Illumina data using Pilon v.1.23 (Walker et al. 2014). The quality of the genome assembly was assessed using Benchmarking Universal Single-Copy Orthologs (BUSCO) v.3.0.2, based on the fungi_odb10 database (Simão et al. 2015) and Core Eukaryotic Genes Mapping Approach (CEGMA) v.2.5 (Parra et al. 2007). Telomeres were manually annotated by identifying tandem repeat sequences (CCCTAA)n or (TTAGGG)n located at the ends of scaffolds (Lee et al. 2021). Transfer RNAs (tRNAs) were detected using tRNAscan-SE v.2.0 (Lowe et al. 1997). Genome annotation was carried out with the MAKER2 v.2.31.9 pipeline (Holt et al. 2011), employing three *ab inito* gene prediction methods: Augustus (Stanke et al. 2006), GeneMark-ES (Lukashin et al. 1998) and SNAP (Korf 2004). Local BLAST searches were performed against the NCBI Non-Redundant Protein Database (Nr) (Sayers et al. 2022), the Kyoto Encyclopedia of Genes and Genomes (KEGG) (Kanehisa et al. 2000) and the Clusters of Orthologous Groups of proteins (COG) (Tatusov et al. 2000) using DIAMOND v.0.8.35 (Buchfink et al. 2021). Additionally, HMMER v.3.1b2 (Eddy 2011) was used for local searches of protein signatures against the Pfam database (Finn et al. 2016). Gene Ontology (GO) (Ashburner et al. 2000) annotations was conducted using Blast2GO v.2.5 (Götz et al. 2008). The upset plot for multi-database annotation was generated using the online tool ChiPlot (https://www.chiplot.online/). Carbohydrate-active enzymes (CAZymes) were identified through the CAZy database (https://www.cazy.org/). Predictions of secondary metabolite gene clusters were made using the web-based fungal version of antiSMASH v.7.0 (Blin et al. 2023). The presence of signal peptides in putative proteins was predicted with SignalP v.4.1 (Petersen et al. 2011) and transmembrane domain predictions was performed using TMHMM v.2.0 (Krogh et al. 2001). Sequences harbouring a signal peptide cleavage site, but lacking a transmembrane region were categorised as putatively secreted proteins.

### ﻿Mating-type locus structure and expression assay

The aforementioned annotation methodology effectively identified *APN2* (encoding a DNA lyase), one of the genes commonly located adjacent to mating-type (*MAT*) genes. However, the *MAT* genes (*MAT1-1-1* and *MAT1-2-1*) and *SLA2* (encoding a cytoskeleton assembly control protein) were not annotated. To locate these genes within the genome of *C.blackwelliae*, local BLAST searches were conducted with corresponding genes of *C.militaris* (GenBank accession nos. KP721256 for *MAT1-1-1*, KP721273 for *MAT1-2-1* and XM_006671662 for *SLA2*) as queries. To assess the expression of the identified *MAT* genes in *C.blackwelliae*, RNA sequencing (RNA-seq) and real-time quantitative reverse transcription PCR (RT-qPCR) analyses were conducted, following the methodologies outlined below.

Structural comparisons at the *MAT* locus were performed across nine additional *Cordyceps* species/strains, including *C.cateniannulata* (FRD 24 and MBC 23), *C.chanhua* (ZJ1611, BA-001 and CC02), *C.fumosorosea* (ARSEF 2679), *C.javanica* (F084), *C.militaris* (ATCC 34164) and *C.tenuipes* (YF17002). All sequences were retrieved from GenBank (Suppl. material 1: table S2) and the *MAT*, *APN2* and *SLA2* genes were identified through local BLAST searches as described above. The results were visualised using IBS v.1.0 (Liu et al. 2015). Amino acid sequences of the *MAT* genes were aligned using the MUSCLE algorithm integrated within MEGA v.11 (Tamura et al. 2021) and the conserved domain of *MAT1-2-1* was predicted via the SMART website (https://smart.embl.de/) (Schultz et al. 1998).

To investigate the evolution of *MAT* genes in *Cordyceps*, nucleotide sequences of *MAT1-1-1*, *MAT1-2-1*, as well as the concatenated sequences of *APN2* and *SLA2*, were used for phylogenetic analyses. The nine *Cordyceps* species/strains mentioned earlier, along with *C.blackwelliae* ZYJ0835, were designated as the ingroup, with *Beauveriaasiatica* ARSEF 4384 serving as the outgroup. Following sequence alignment by MUSCLE (Edgar 2004), poorly aligned regions were removed by trimAl (Capella-Gutiérrez et al. 2009). Phylogenetic relationships were estimated using the Maximum-Likelihood method as implemented in IQ-TREE v.1.6.12 (Nguyen et al. 2015) with 1,000 bootstrap replicates. The resulting tree files were visualised using FigTree v.1.4.4 (Rambaut 2009).

### ﻿Phylogenomic analysis

Two datasets were used for phylogenomic analyses. Dataset I comprised 3,488 single-copy nuclear genes, which were extracted using BUSCO v.5.8.0 with the hypocreales_odb10 database (Seppey et al. 2019). Dataset II consisted of 14 core mitochondrial protein-coding genes (i.e. *atp6*, *8-9*, *cob*, *cox1-3*, *nad1-6* and *nad4L*). For dataset I, nuclear phylogenomic matrices were generated using the BUSCO_phylogenomics pipeline (accessible at https://github.com/jamiemcg/BUSCO_phylogenomics). The analysed fungal taxa are detailed in Suppl. material 1: table S3. A Maximum-Likelihood phylogenetic tree was constructed, based on concatenated sequences of 2,039,507 amino acid positions with the method described above. For dataset II, fungal species from genera consistent with those used in dataset I were selected (Suppl. material 1: table S4). Phylogenetic relationships were estimated using a Maximum-Likelihood approach, based on the concatenated sequences of 4,274 amino acid positions using the aforementioned methodology.

### ﻿Transcriptomic analysis

Complementary DNA (cDNAs) libraries were constructed by enriching mRNA from total RNA using polyT oligo-attached magnetic beads and then reverse-transcribed into cDNA. Subsequently, the cDNA libraries were sequenced by Novogene Technology Co., Ltd. (Beijing, China) on an Illumina NovaSeq platform with PE150 reads. Raw sequencing data underwent filtration using fastp v.0.23.0 (Chen et al. 2018) and then clean reads were aligned to the *C.blackwelliae* genome using HISAT2 v.2.0.5 (Kim et al. 2015). The mapped reads for each sample were subsequently assembled using StringTie v.1.3.3b (Pertea et al. 2015). The number of reads mapped to each gene was quantified using FeatureCounts v.1.5.0-p3 (Liao et al. 2014), which facilitated the calculation of gene expression levels represented as fragments per kilobase exon model per million mapped reads (FPKM). Differential expression analysis amongst sample groups was performed using the R package DESeq2 v.1.20.0 (Love et al. 2014), with *P*-values adjusted using the Benjamini and Hochberg’s approach (Benjamini et al. 2018) for controlling the false discovery rate. Genes with an adjusted *P*-value of less than 0.05 and a |log2FoldChange| greater than 2 were regarded as DEGs. GO and KEGG enrichment analyses were performed using clusterProfiler v.3.8.1 (Yu et al. 2012), with an adjusted *P*-value threshold of less than 0.05 deemed statistically significant.

Alternative splicing (AS) events were analysed using rMATS v.4.1.0 (Shen et al. 2014). Various types of AS events, including exon skipping (SE), mutually exclusive exon (MXE), intron retention (RI), alternative 5’ splicing (A5SS) and alternative 3’ splicing (A3SS), were compared across different growth stages, with a false discovery rate (FDR) < 0.05 indicating statistical significance. The significant AS events were visualised using the Python programme rmats2sashimiplot (https://github.com/Xinglab/rmats2sashimiplot). Additionally, GO enrichment analysis was performed on all genes associated with the identified AS events using the aforementioned methodology.

### ﻿Confirmation of RNA-seq data by RT-qPCR

To confirm the reliability of RNA-seq data, four DEGs (*gene 6087*, *gene 0120*, *gene 1814* and *gene 1408*) and two non-DEGs (*gene 5222* and *gene 0234*) were randomly selected and their expression levels were quantified through RT-qPCR. Complementary DNA were synthesised from 100 ng of mRNA, enriched as described above, using the *TransScript*^®^ One-Step gDNA Removal and cDNA Synthesis SuperMix (TransGen Biotech, Beijing, China) in a 20 μl reaction mixture that included 1 μl of *TransScript*^®^ RT/RI Enzyme Mix, 10 μl of 2×TS Reaction Mix, 1 μl of Anchored Oligo(dT)_18_ Primer, 1 μl of gDNA Remover, 2 μl of RNase-free water and 5 μl of mRNA. RNA concentration and purity (i.e. OD260/OD280) were measured using NanoDrop^TM^ One Microvolume UV-Vis Spectrophotometers (Thermo Scientific, USA). RT-qPCR was performed on a CFX Connect^TM^ Real-Time PCR system (Bio-Rad, USA). The RT-qPCR reaction mixture (in 20 μl) consisted of 10 μl of 2× PerfectStart^®^ Green qPCR SuperMix, 0.4 μl of each primer (10 μM), 3 μl of diluted cDNA (200 ng/μl) and 6.2 μl of nuclease-free water. The RT-qPCR programme included an initial denaturation step at 95°C for 30 s, followed by 40 cycles of 10 s at 95°C, 20 s at 56°C and 15 s at 72°C. All reactions were conducted in biological triplicates. Fluorescence signals were recorded during the extension stage and a melting curve was set to detect the specificity of the amplified products. Relative gene expression was evaluated using the 2^-ΔΔCT^ method (Livak et al. 2001). The 18S rRNA was utilised as an internal reference, which has been previously validated as a stable internal reference gene in *C.blackwelliae* (Li et al. 2025b). All primers used in this study are listed in Suppl. material 1: table S5. Least significance difference (LSD) test was used to assess the statistical significance of differences observed across different stages.

### ﻿Weighted gene co-expression network analysis (WGCNA)

The R package WGCNA was employed to conduct a weighted correlation analysis based on all genes (Langfelder et al. 2008). An adjacency matrix was calculated with the optimal soft-thresholding power (β) (Suppl. material 2: fig. S1) and transformed into a topological overlap matrix (TOM), which was further converted into a distance matrix to build a tree of gene clusters. A dynamic cutting algorithm was applied to cluster all genes into colour-coded modules. To identify modules that are strongly related to phenotypic traits, Pearson’s correlation coefficients (Benesty et al. 2009) were calculated between each module and each sample. A heat map was drawn according to these correlation coefficients, allowing for the selection of phenotype-related modules. A gene co-expression network was constructed, with a focus on edges with a weight exceeding 0.2 in the magenta and black modules and a weight greater than 0.36 in the turquoise module. The resulting network was visualised using Cytoscape 3.10.2 (Shannon et al. 2003). Hub genes were identified as the most highly connected nodes within each module.

### ﻿Metabolomic analysis

Samples of LM, WM and FB were subjected to grinding in liquid nitrogen to produce fine powders suitable for Ultra-High-Performance Liquid Chromatography coupled with Tandem Mass Spectrometry (UHPLC-MS/MS) analysis. This analysis was conducted using a Vanquish UHPLC system (Thermo Fisher, Germany) in conjunction with an Orbitrap Q Exactive^TM^ HF mass spectrometer (Thermo Fisher, Germany). Samples were injected on to a Hypersil Gold column (100 × 2.1 mm, 1.9 μm) using a 12-min linear gradient at a flow rate of 0.2 ml/min. For a positive polarity mode, analyses were performed with (A) 0.1% formic acid in water and (B) methanol. For a negative polarity mode, mobile phases comprised (A) 5 mM ammonium acetate (pH = 9.0) and (B) methanol. The separation process followed the gradient: 2% B, 1.5 min; 2%–85% B, 3 min; 85%–100% B, 10 min; 100%–2% B, 10.1 min; and 2% B, 12 min. The Q Exactive^TM^ HF mass spectrometer was operated in positive/negative polarity mode with a spray voltage set at 3.5 KV, a capillary temperature of 320°C, a sheath gas flow rate of 35 psi, an aux gas flow rate of 10 l/min, an S-lens RF level of 60 and an aux gas heater temperature of 350°C.

The raw data files obtained from UHPLC-MS/MS were processed using Compound Discoverer 3.3 (Thermo Fisher, Germany) for data pretreatments, which included peak alignment, peak feature extraction and quantification. Subsequently, the identified peaks were matched against mzCloud (https://www.mzcloud.org/), mzVault (Thermo Fisher, Germany) and MassList (Novogene, China) databases. Statistical analyses were performed using R v.3.4.3 and Python v.2.7.6. The metabolites were annotated using HMDB (https://hmdb.ca/metabolites) and LIPIDMaps (http://www.lipidmaps.org/) databases. Partial Least Squares Discriminant Analysis (PLS-DA) was performed using metaX (Westerhuis et al. 2010; Wen et al. 2017). Univariate analysis (*t*-test) was applied to calculate the statistical significance (*P*-value). Metabolites were considered as DMs if they exhibit a variable importance in projection (VIP) > 1, a *P*-value < 0.05 and a fold change (FC) ≥ 2 or FC ≤ 0.5.

### ﻿Conjoint analysis of transcriptomics and metabolomics

To investigate the correlation between DEGs and DMs, Pearson’s correlation coefficient between gene expression and metabolite abundance were calculated using the mixOmics package in R (Rohart et al. 2017). The top 50 DMs and top 50 DEGs were presented, based on their statistical significance (*P*-value). A correlation heatmap illustrating the relationship between genes and metabolites was built using the corrplot package in R (Wei et al. 2017). Additionally, KEGG co-enrichment diagrams were created by utilising the ggplot2 package in R (Wickham. 2009). The global metabolic pathway was visualised through iPath v.3.0. (https://pathways.embl.de/).

## ﻿Results

### ﻿Assembly and annotation of the *C.blackwelliae* genome

To establish a reference genome for *C.blackwelliae*, Illumina and Nanopore sequencing technologies were employed to generate short and long reads, respectively. These sequencing efforts yielded 6.55 Gb of Illumina data (21,697,168 paired reads, 211× coverage) and 5.37 Gb of Nanopore data (623,214 reads, 173× coverage). Except for a single scaffold (i.e. Scaffold10) representing the mitochondrial genome, the nuclear genome was assembled into 11 scaffolds, totally 31.06 Mb, with an N50 value of 4.14 Mb (Table 1). Notably, telomeric repeats were identified at both termini of three scaffolds (i.e. scaffolds 2, 7 and 8) and at either the 5’ or 3’ end of five scaffolds (i.e. scaffolds 1, 3, 9, 11 and 12) (Suppl. material 1: table S6). BUSCO and CEGMA analyses confirmed that the assembly captured 99.3% of fungal single-copy orthologs and 95.6% of core eukaryotic genes, respectively (Table 1).

**Table 1.** Statistics of genome assembly and gene prediction of *C.blackwelliae* ZYJ0835.

Item	Value	Item	Value
Genome size (bp)	31,062,909	No. of putative genes	8,138†
Sequence coverage	>173×	Total length of protein-coding genes (bp)	19,606,463
No. of scaffolds	11	Gene average length (bp)	2256.47
Largest scaffold size (bp)	7,729,812	Gene density (per Mb)	280
N50 (bp)	4,140,478	No. of tRNA	135
GC content (%)	53.86	Repeat rate (%)	0.05
Complete BUSCOs (%)	99.30	No. of secondary metabolite clusters	41
Missing BUSCOs (%)	0.70	No. of CAZymes	255
Complete CEGMA (%)	95.56	No. of secreted proteins	713

Note: †This number comprises 8,136 originally predicted genes, supplemented by two additional genes (*MAT1-2-1* and *SLA2*) identified via BLAST searches. Notably, the truncated gene *MAT1-1-1* is excluded from the count due to its incomplete nature. All subsequent analyses presented in this table (e.g. secondary metabolite clusters and CAZymes) are based exclusively on the 8,136 originally predicted genes.

A total of 8,136 genes were predicted, including 7,969 protein-coding genes, 129 tRNA genes and 38 rRNA genes. Functional annotations using NR, GO, KEGG, COG and Pfam databases revealed that 1,585 genes were annotated across all databases and 6,435 genes had annotation in at least two databases (Suppl. material 2: fig. S2; Suppl. material 1: table S7). The most enriched GO terms were related to “Cellular anatomical entity”, “Catalytic activity” and “Binding” (Suppl. material 2: fig. S3a). These annotated genes accounted for 54.7% of the total protein coding genes and are likely to play a significant role in developmental processes. The top three COG categories were “Carbohydrate transport and metabolism”, “General function prediction only” and “Translation, ribosomal structure and biogenesis”, which encompassed 351, 326 and 294 genes, respectively (Suppl. material 2: fig. S3b). KEGG annotation assigned 3,126 proteins to 387 distinct pathways, categorised into seven major functional groups: cellular processes (5 branches, 416 genes), environmental information processing (3 branches, 229 genes), genetic information processing (4 branches, 610 genes), human diseases (12 branches, 524 genes), metabolism (12 branches, 847 genes), organismal systems (10 branches, 363 genes) and 1,194 unclassified genes. Amongst the annotated genes, the category “Global and overview maps” contained the highest number of genes, followed by “Transport and catabolism”, “Signal transduction”, “Translation” and “Carbohydrate metabolism” (Suppl. material 2: fig. S3c).

### ﻿Expression and evolution of mating-type genes

**Figure 1.**
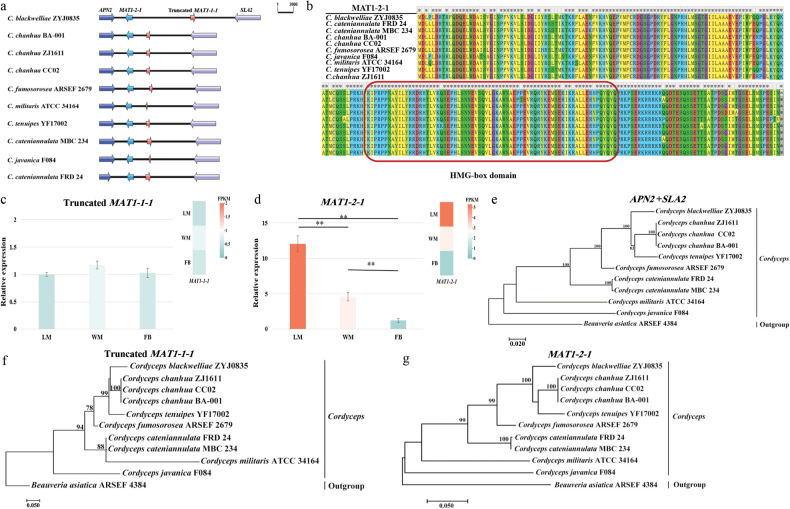
Structural and evolutionary analysis of the *MAT* locus in *C.blackwelliae*: **a** schematic representation of the *MAT1-2* locus across various *Cordyceps* species/strains. The *APN2* gene, which encodes a DNA lyase and the *SLA2* gene, which encodes a protein involved in cytoskeleton assemble control, flank the *MAT* genes. Gene orientations are indicated by arrows. Please refer to Suppl. material 1: table S2 for details of fungal strains and corresponding scaffolds harbouring *MAT*, *APN2* and *SLA2* genes; **b** alignment of amino acid sequences of the MAT1-2-1 protein, with the conserved HMG-box domain indicated by a red box; **c, d** validation of *MAT* gene expression at three different developmental stages through RT-qPCR analysis. Relative expression was calculated using the 2^-ΔΔCT^ method and normalised against the LM stage of the truncated *MAT1-1-1* gene. Gene expression levels (FPKM) from RNA-seq data were presented as heatmaps **e–g** phylogenetic analyses based on the concatenated *SLA2* and *APN2* genes (**e**), the truncated *MAT1-1-1* gene (**f**) and the *MAT1-2-1* gene (**g**).

To investigate *C.blackwelliae* fruiting body formation, the *MAT* locus structure was analysed. The genome contained a complete *MAT1-2-1* gene and a truncated *MAT1-1-1* gene, a pattern conserved across ten *Cordyceps* genomes (Fig. 1a). The two *MAT* genes with opposite transcriptional orientations were found to be more distantly spaced in *C.blackwelliae* (7.3 kb) than those in other *Cordyceps* species/strains (1.5–2.1 kb). The *MAT1-2-1* gene within these *Cordyceps* genomes contained two introns (46–51 bp) and the amino acid sequence alignment revealed an 85.36% conservation rate amongst different *Cordyceps* species/strains (Fig. 1b). The HMG-box domain was identified through searches in the SMART database, with strong *E*-values (1.53e−17 to 8.76e−18).

To further clarify the potential functions of *MAT1-2-1* and the truncated *MAT1-1-1* gene in the regulation of fruiting body formation, gene expression profiles were examined across three different developmental stages. The *MAT1-2-1* gene exhibited differential expression (read counts 0–122 and FPKM values 0–8.67), with the lowest expression levels observed at the FB stage, while expression levels increased by 8.2-fold and 4.2-fold at the LM and WM stages, respectively (Suppl. material 1: table S8). In contrast, the truncated *MAT1-1-1* gene demonstrated minimal expression (read counts 0–11 and FPKM values 0–1.41) throughout the three developmental stages (Suppl. material 1: table S8). RT-qPCR confirmed these expression patterns (Fig. 1c, d).

To ascertain whether the different *MAT* genes exhibit divergent evolutionary trajectories, phylogenetic analyses were conducted, based on nucleotide sequences of *MAT1-2-1*, the truncated *MAT1-1-1* or concatenated sequences of *SLA2* and *APN2*. The topologies of the three phylogenetic trees were found to be basically congruent, consistently placing *C.blackwelliae* in close relationship to *C.chanhua* and *C.tenuipes* (Fig. 1e–g).

### ﻿Phylogenomic analyses based on nuclear and mitochondrial genomes

**Figure 2.**
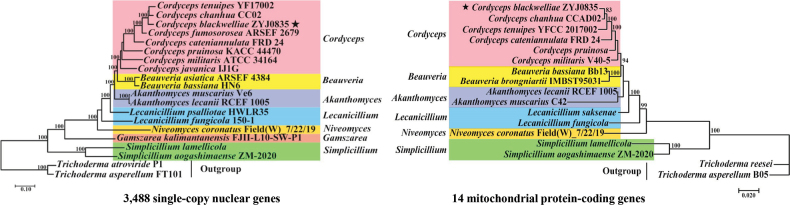
Phylogenomic analyses based on both nuclear and mitochondrial genomes. The nuclear phylogeny (left) was constructed, based on 3,488 single-copy orthologs, while the mitochondrial phylogeny (right) was constructed, based on 14 core protein-coding genes. Detailed information regarding the fungal taxa and their corresponding accession numbers can be found in Suppl. material 1: tables S3, S4. The target fungus of this study is marked with a star (black star).

To elucidate the phylogenetic placement of *C.blackwelliae* within the family *Cordycipitaceae*, phylogenetic trees were constructed, based on 3,488 nuclear single-copy orthologs and 14 mitochondrial core protein-coding genes, respectively. Both analyses robustly clustered *Cordyceps* species together (Fig. 2). Each of the other genera that included more than two taxa formed distinct clades, with the exception of *Lecanicillium*. Both phylogenetic trees consistently positioned *C.blackwelliae* in close phylogenetic proximity to *C.chanhua* and *C.tenuipes*, although some minor discrepancies were noted (Fig. 2). The mitochondrial phylogeny positioned *C.blackwelliae* closely with *C.chanhua*, followed by *C.tenuipes*, while the nuclear phylogeny grouped *C.chanhua* and *C.tenuipes* together, with *C.blackwelliae* as their closest relative.

### ﻿Gene expression profiling across different developmental stages

To elucidate the molecular mechanisms underlying fruiting body development, a total of 15 RNA-seq libraries were generated from three growth stages (i.e. LM, WM and FB), each with five biological replicates. The sequencing process yielded 631 million raw reads; following quality control and cleaning procedures, 601 million clean reads were retained, resulting in an average of 40.1 million reads per replicate (Suppl. material 1: table S9). Notably, more than 90% clean reads were successfully mapped to the *C.blackwelliae* genome and 553 novel genes were identified (Suppl. material 1: table S10). Principal component analysis (PCA) demonstrated clear separation amongst stages and strong replicate correlations (Suppl. material 2: fig. S4a).

**Figure 3.**
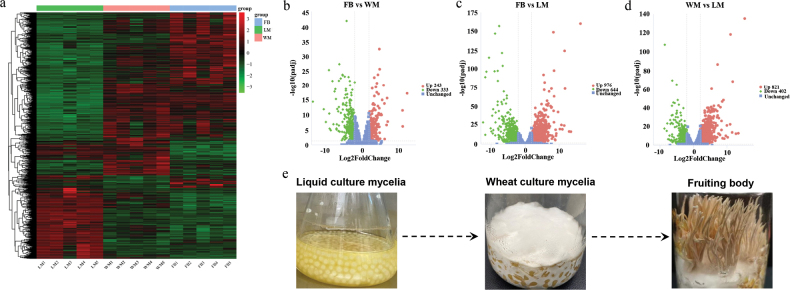
Gene expression profiles across three developmental stages: **a** clustering heat map of DEGs. Each column represents a sample, and each row corresponds to a gene. The FPKM values are indicated by a colour gradient, with red indicating high expression and green indicating low expression. The results of gene clustering are displayed as the dendrograms on the left; **b–d** volcano plot of DEGs between different developmental stages, with red dots indicating up-regulated genes, green dots indicating down-regulated genes and blue dots representing genes without statistically significant expression variation. The two vertical dashed lines represent |log2FoldChange| of 2, while the horizontal dashed lines represent the Padj value thresholds of 0.05; **e***C.blackwelliae* samples at three different developmental stages that were used to perform RNA-seq and metabolomic analyses.

To identify genes that may play a crucial role in mycelial growth and fruiting body development, three comparative analyses were conducted: WM vs. LM, FB vs. WM and FB vs. LM. The analyses revealed that 2,078 genes exhibited differential expression with a |log2FoldChange| > 2 (Suppl. material 1: table S11), with 121 DEGs shared across all three comparisons (Suppl. material 2: fig. S4b). Heatmap clustering showed that the highest number of DEGs was observed in the FB vs. LM comparison, while the lowest was noted in the FB vs. WM comparison (Fig. 3a). Transcription profiles of DEGs were visualised through volcano plots utilising -log10(padj) and log2(FoldChange) as statistical parameters (Fig. 3b–d), highlighting the most significantly regulated genes. Notably, *MetB* (*gene 6635*), encoding a putative cystathionine gamma-synthase involved in Cys/Met metabolism, was not expressed at the FB stage, but active at both LM and WM stages (FPKM values 4.6–314.85). At the LM stage, *gene 4153*, encoding a putative HSP20-like chaperone, was dramatically up-regulated by 385-fold compared to the WM stage and 466-fold compared to the FB stage. Conversely, *gene 6087*, encoding a putative RNA binding protein, was significantly down-regulated by 38,736-fold compared to the WM stage and 77,560-fold compared to the FB stage (Suppl. material 1: table S12). In addition, ribosomal genes were significantly up-regulated during the WM stage, while septin protein family genes were up-regulated during the FB stage. Furthermore, genes encoding chitin deacetylase and β-1,3-glucanase, related to cell wall remodelling, were up-regulated during the FB stage, reflecting dynamic cell wall modifications essential for fruiting body development (Suppl. material 2: fig. S5). To validate the RNA-seq results, four DEGs and two non-DEGs were randomly chosen for validation by RT-qPCR. The RT-qPCR results showed strong concordance with the RNA-seq data, confirming the reliability of our transcriptomic analysis (Suppl. material 2: fig. S6).

To identify pathways associated with phenotypes, GO and KEGG enrichment analysis was performed on the 2,078 DEGs. The findings indicated a significant overexpression of genes linked to DNA repair and recombination, including those involved in “Base excision repair”, “Mismatch repair” and “Homologous recombination”. This overexpression is crucial for maintaining genomic stability during rapid cell division and growth, particularly at the LM stage, where the mycelia exhibited rapid growth, forming dense and homogeneous mycelial pellets (Fig. 3e). Furthermore, the up-regulated expression of membrane-related GO terms, including “Integral component of membrane”, “Membrane parts” and “Transmembrane transporter activity”, also occurs at the LM stage. This up-regulation is crucial for maintaining cellular integrity, as well as for facilitating intercellular communication and signal transduction, supporting the observed rapid proliferation and biomass accumulation (Fig. 3e). At the WM stage, the up-regulated genes were significantly enriched in pathways associated with endopeptidase, oxidoreductase, peptidase and hydrolase activities. Additionally, the up-regulation of co-factor binding functions, including iron ion, heme and tetrapyrrole binding, is crucial for the proper functioning of many enzymes, as these co-factors are involved in electron transfer and catalytic reactions. This coordinated regulation of enzyme activity and co-factor binding likely supports the decomposition and utilisation of nutrients in the culture medium by the vegetative mycelia, which spread across the solid medium surface and penetrate the substrate to form an extensive hyphal network. At the FB stage, there was a significant up-regulation of genes involved in various biosynthesis and catabolism processes, including those related to amino acids, N-glycan, sphingolipids, carbohydrates, glycerophospholipids and nucleotides (Suppl. material 1: table S13). These processes are likely to be pivotal in the development of fruiting bodies and signify the transition from vegetative to reproductive growth, characterised by the differentiation of mycelia into specialised structures such as stalks and spore-producing tissues (Fig. 3e).

Alternative splicing (AS) events were examined across the three developmental stages (Suppl. material 1: table S14). A total of 707 AS events were identified in the FB vs. LM comparison, comprising 670 SE and 37 MXE. In the FB vs. WM comparison, we observed 658 AS events, consisting of 623 SE and 35 MXE. The WM vs. LM comparison revealed 704 AS events, with 668 SE and 36 MXE. Notably, we found no evidence of RI, A5SS and A3SS. Considering that several genes exhibited two or more AS events, we estimated that a total of 681 genes underwent AS with each gene being counted only once. Of particular interest, 53.23% of SE and 23.91% of MXE were present in all three comparisons. These findings indicated that AS is dynamically regulated during development. Nine significant AS events were identified according to our stringent criteria (Suppl. material 2: fig. S7, Suppl. material 1: table S14). GO annotation analysis revealed that 40 genes, associated with the “Structural molecule activity” term, exhibited alternative splicing, accounting for 27% of all genes categorised under this GO term. This was followed by “Transporter activity” and “ATP-dependent activity”, which also demonstrated a high frequency of AS events and a great proportion of affected genes (Suppl. material 2: fig. S8).

### ﻿WGCNA investigation of development-related module genes

**Figure 4.**
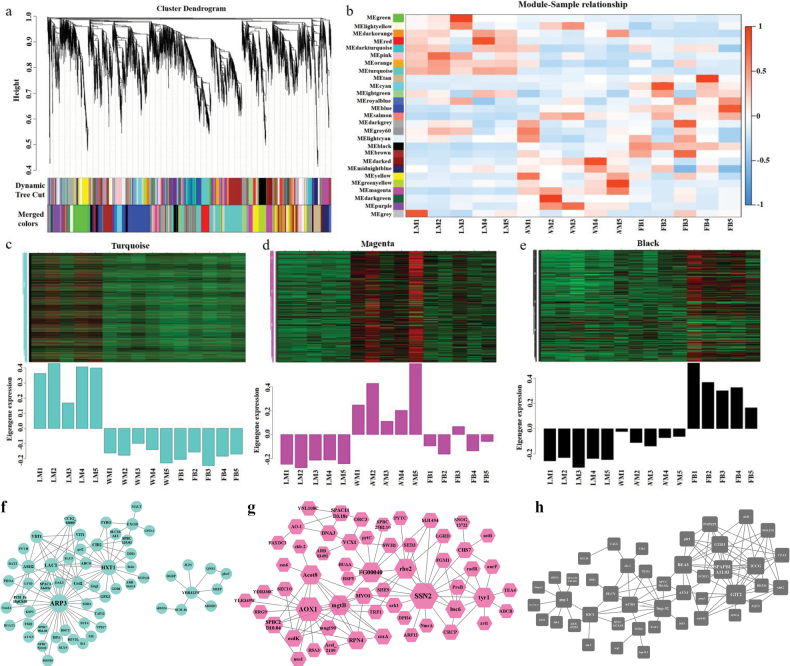
WGCNA investigation of modules associated with growth and fruiting body development: **a** display of original and merged modules within the clustering tree. The upper section of the diagram illustrates the gene clustering tree within the network. Each branch of the hierarchical tree or each vertical line in the accompanying colour bar corresponds to an individual gene. The middle section represents the original modules generated through dynamic tree clipping. The merged colours at the bottom indicate merged modules with a dissimilarity coefficient of less than 0.25. Genes not assigned to any modules are depicted in grey; **b** heatmap of correlations between modules and samples as indicated by a colour gradient, with red indicating a positive correlation and blue indicating a negative correlation between modules and samples. The turquoise, magenta and black modules are positively correlated with the LM, WM and FB stages, respectively; **c–e** gene expression patterns of the turquoise (**c**), magenta (**d**) and black (**e**) modules, with specific gene information available in Suppl. material 1: table S15; **f–h** gene co-expression networks corresponding to the turquoise (**f**), magenta (**g**) and black (**h**) modules. The construction of the gene co-expression network for each module involved selecting edges, based on weight thresholds of 0.36 for the turquoise module and 0.20 for both the black and magenta modules. The turquoise module comprises 58 nodes and 101 edges, the magenta module includes 56 nodes and 101 edges and the black module consists of 44 nodes and 95 edges. In this context, nodes represent individual genes, while edges represent interactions between gene pairs.

WGCNA was utilised to construct a gene co-expression network and to identify hub genes implicated in fungal development, based on 8,136 initially predicted genes and 553 novel genes. The analysis revealed 26 distinct gene modules (Fig. 4a). To identify modules strongly correlated with phenotypes, a correlation heatmap of modules and samples was generated (Fig. 4b). Based on the module-sample relationships, three modules were selected: a turquoise module (1,610 genes) that showed peak expression at the LM stage (Fig. 4c), a magenta module (333 genes) with positive correlation to the WM stage (Fig. 4d) and a black module (396 genes) that was overexpressed at the FB stage (Fig. 4e). These modules were used to construct the gene co-expression network (Suppl. material 1: table S15; Fig. 4f–h). The network revealed the following hub genes: at the LM stage—*ARP3* (actin-like protein 3), *HXT1* (low-affinity glucose transporter), *LAC1* (L-ascorbate oxidase) and *CIR2* (electron transfer flavoprotein-ubiquinone oxidoreductase) (Fig. 4f); at the WM stage—*SSN2* (RNA polymerase II mediator subunit), *AOX1* (alternative oxidase) and *CHS7* (Chitin synthase III catalytic subunit) (Fig. 4g); and at the FB stage—*gene 4088* (alpha-hydroxy acid dehydrogenase), *GIT2* (glycerophosphodiester transporter) and *BEA3* (ABC transporter), all likely involved in fruiting body formation (Fig. 4h). Notably, the occurrence of AS events in these hub genes (27.27%) was significantly higher than in the remaining genes (7.84%)

### ﻿Metabolomic profiling across different developmental stages

In order to elucidate the metabolic dynamics associated with morphogenesis and maturation, an analysis utilising UHPLC-MS/MS was performed. A comparative assessment of the total ion chromatograms indicated that, although the types of metabolites present across the three developmental stages were comparable, there were notable differences in the concentrations (Suppl. material 2: fig. S9). A total of 692 metabolites were identified in the positive ion mode and 469 metabolites in the negative ion mode (Suppl. material 1: table S16). These metabolites were classified into ten distinct categories, with lipids and lipid-like molecules making up the largest fraction (39.41%), followed by organic acids and their derivatives (21.19%) (Suppl. material 2: fig. S10a). Amongst the identified lipids and lipid-like molecules, the predominant subclasses included fatty acids and their conjugates, glycerophosphoserines and eicosanoids (Suppl. material 2: fig. S10b).

To characterise the global metabolic differences across the three developmental stages, PLS-DA was conducted. The first two principal components of the PLS-DA score plot accounted for over 70% of the total variance, clearly distinguishing between developmental stages. Additionally, the R^2^Y and Q^2^Y values approached one, confirming the model’s accuracy and reliability (Suppl. material 2: fig. S11a–c). A permutation test was conducted to evaluate the model’s quality, revealing that all red Q^2^ and blue R^2^ values on the left were lower than the original points on the right (Suppl. material 2: fig. S11d–f), which suggests that the model is not subject to overfitting. A total of 1,014 DMs were identified across the three stages (Suppl. material 2: fig. S12). Notably, amino acids were significantly more abundant at the FB stage, likely playing a critical role in the biosynthesis of structural proteins, enzymes and other nitrogen-containing compounds essential for fruiting body development (Suppl. material 2: fig. S13). Conversely, corey lactone diol (a gamma-butyrolactone) was more abundant in the mycelial stages (i.e. LM and WM), particularly at the WM stage and less so at the FB stage. Previous studies have shown that butyrolactones can increase hyphal branching and reduce hyphal extension in fungi like *Aspergillusterreus* (Schimmel et al. 1998). Thus, this metabolite likely promotes dense mycelial network formation during the mycelial stage, enhancing nutrient absorption and substrate colonisation (Suppl. material 2: fig. S14).

### ﻿Conjoint analysis of transcriptomics and metabolomics

**Figure 5.**
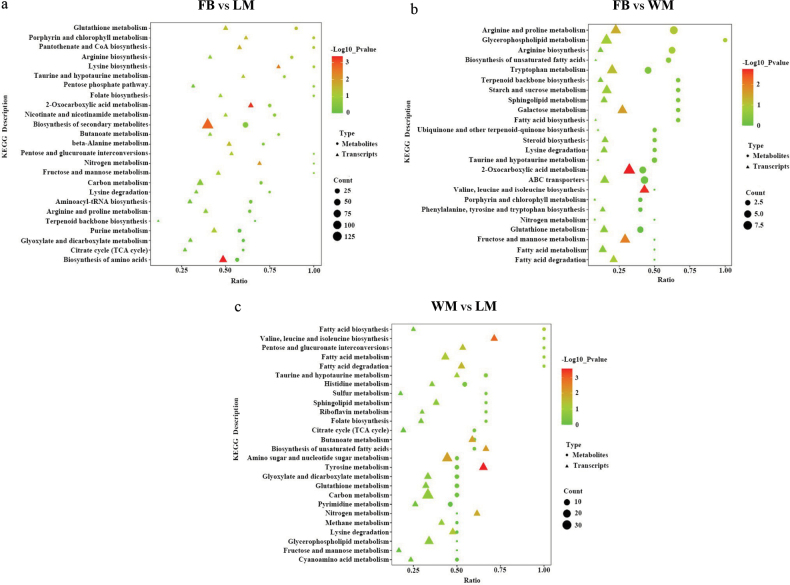
KEGG pathway enrichment analysis of DEGs and DMs across the three distinct comparisons. The horizontal axes indicate gene ratios, which reflect the proportion of DEGs/DMs relative to the total number of genes/metabolites associated with a specific KEGG pathway. The size of the data points corresponds to gene counts and the colour represents the -log10 *P*-value.

To better understand the pivotal genes and metabolites implicated in the differentiation of fruiting bodies, we analysed the similarities in gene expression and metabolite profiles across the three developmental stages. KEGG co-enrichment analysis revealed that both DMs and DEGs were significantly enriched in the pathways of “Nitrogen metabolism” and “Lysine biosynthesis” when comparing FB to LM (Fig. 5a). In the FB vs. WM comparison, the pathways enriched for both DMs and DEGs included “Starch and sucrose metabolism” and “Biosynthesis of unsaturated fatty acids” (Fig. 5b). Additionally, “Tyrosine metabolism” and “Butanoate metabolism” were enriched in the WM vs. LM comparison (Fig. 5c).

For a comprehensive understanding of the transitions associated with fruiting body differentiation, DEGs and DMs from the FB vs. WM comparison were mapped on to the metabolic pathways using iPath. The results were consistent with the GO and KEGG enrichment analyses of DEGs, indicating that pathways related to purine, amino sugar, nucleotide sugar, glycerophospholipid, starch, sucrose, phenylalanine and steroid metabolism and biosynthesis are essential for fruiting body development (Suppl. material 2: fig. S15a). Notably, at the FB stage, pathways for galactose, fructose, mannose, starch and sucrose metabolism were markedly up-regulated. The hydrolysis of macromolecular cellodextrin yielded sucrose and α,α-trehalose, which are readily utilised to provide energy for fruiting body formation (Suppl. material 2: fig. S15b). In contrast, metabolic pathways related to arachidonic acid, linoleic acid and α-linolenic acid metabolism were down-regulated at the FB stage (Suppl. material 2: fig. S15c).

**Figure 6.**
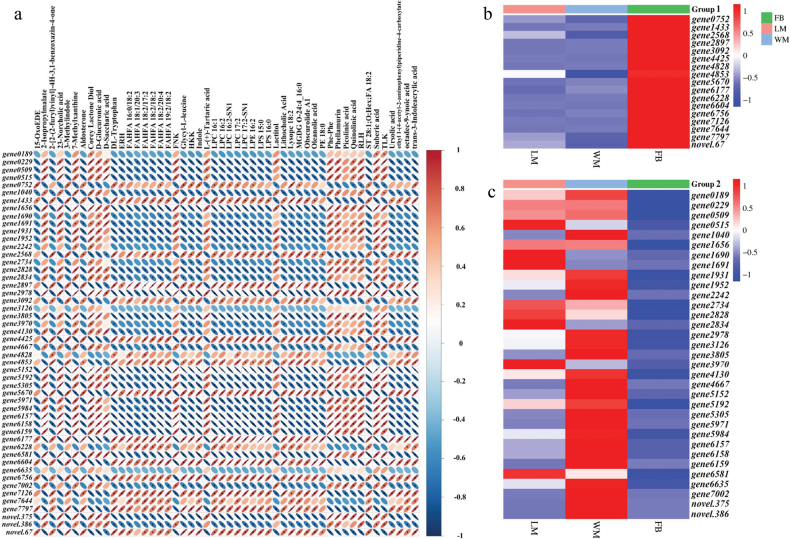
Correlation analysis between DEGs and DMs in the FB vs. WM comparison: **a** the figure shows the pairwise correlations between the top 50 DMs and the top 50 DEGs, sorted by *P*-values from the smallest to the largest. The intensity of the colour indicates the strength of the correlation, with red hues representing strong positive correlations and blue hues indicating strong negative correlations. Correlation values are more pronounced when the ellipses are flatter and statistically significant correlations (*P* < 0.05) are denoted with an asterisk (*); **b, c** based on the results shown in panel a, metabolite-regulating genes were categorised into two groups. Group 1 comprises 17 genes that show a positive correlation with 34 metabolites (Table 2), but a negative correlation with the remaining 16 metabolites (**b**). In contrast, Group 2 genes exhibit an inverse regulatory pattern relative to Group 1 (**c**).

Furthermore, a correlation plot was generated to illustrate the relationship between DEGs and DMs in the FB vs. WM comparison. This analysis identified two distinct groups of genes regulating metabolites. Group 1 consisted of 17 genes (including *gene 0752* and *gene 1433*), exhibiting a similar regulatory pattern, while Group 2 contained the remaining 33 genes displaying an opposing regulatory pattern (Fig. 6a). Group 1 genes were significantly up-regulated at the FB stage (Fig. 6b), whereas Group 2 genes were down-regulated (Fig. 6c). Consequently, the regulation of fruiting body development was predominantly influenced by Group 1 genes. A total of 34 metabolites, positively correlated with Group 1 genes, showed significant increases at the FB stage (Table 2; Suppl. material 2: fig. S16), suggesting their crucial roles in fruiting body development.

**Table 2.** Metabolites significantly related to fruiting body development according to transcriptomic and metabolomic correlation analysis.

**Metabolite name**	**Formula**	**Class**	**log2FC**†	***P*-value**	**VIP**‡
LPC 16:2§	C_24_H_46_NO_7_P	Lipids and lipid-like molecules	4.353	2.19E^-13^	1.792
LPC 17:2	C_25_H_48_NO_7_P	Lipids and lipid-like molecules	5.015	7.56E^-12^	1.840
MGDG O-24:4_16:0|	C_49_H_88_O_9_	Lipids and lipid-like molecules	6.441	1.65E^-11^	1.639
Trans-3-Indoleacrylic acid	C_11_H_9_NO_2_	Organoheterocyclic compounds	1.863	5.18E^-11^	1.764
Indole	C_8_H_7_N	Organoheterocyclic compounds	1.497	1.51E^-10^	1.767
LPC 17:2-SN1	C_25_H_48_NO_7_P	Lipids and lipid-like molecules	3.635	1.82E^-10^	1.702
FAHFA 16:0/18:2¶	C_34_H_62_O_4_	Lipids and lipid-like molecules	5.684	1.99E^-10^	1.652
Lithocholic Acid	C_24_H_40_O_3_	Lipids and lipid-like molecules	3.642	7.27E^-10^	1.828
DL-Tryptophan	C_11_H_12_N_2_O_2_	Organoheterocyclic compounds	2.023	1.43E^-09^	1.271
LPC 16:2-SN1	C_24_H_46_NO_7_P	Lipids and lipid-like molecules	3.806	2.95E^-09^	1.752
FAHFA 18:2/18:2	C_36_H_62_O_4_	Lipids and lipid-like molecules	6.151	3.67E^-09^	1.675
FAHFA 18:2/17:2	C_35_H_60_ O_4_	Lipids and lipid-like molecules	5.266	6.21E^-09^	1.719
LPC 16:1	C_24_H_48_NO_7_P	Lipids and lipid-like molecules	5.464	2.92E^-08^	1.817
HKK#	C_18_H_33_N_7_O_4_	Organic acids and derivatives	2.706	3.57E^-08^	1.728
LPE 16:2††	C_21_H_40_NO_7_P	Lipids and lipid-like molecules	2.545	3.86E^-08^	1.702
3-Methylindole	C_9_H_9_N	Organoheterocyclic compounds	1.337	4.47E^-08^	1.708
2-[2-(2-furyl)vinyl]-4H-3,1-benzoxazin-4-one	C_14_H_9_NO_3_	Organoheterocyclic compounds	4.895	5.23E^-08^	1.810
FAHFA 18:1/20:3	C_38_H_66_O_4_	Lipids and lipid-like molecules	6.412	9.61E^-08^	1.533
Aldosterone	C_21_H_28_O_5_	Lipids and lipid-like molecules	2.282	1.04E^-07^	1.791
Ethyl 1-(4-acetyl-2-aminophenyl) piperidine-4-carboxylate	C_16_H_22_N_2_O_3_	Organoheterocyclic compounds	4.980	1.08E^-07^	1.687
Obscurolide A1	C_15_H_17_NO_5_	Benzenoids	4.535	1.13E^-07^	1.795
LPS 16:0‡‡	C_22_H_44_NO_9_P	Lipids and lipid-like molecules	8.025	1.58E^-07^	1.831
FAHFA 18:2/20:4	C_38_H_62_O_4_	Lipids and lipid-like molecules	2.577	1.63E^-07^	1.368
Ursolic acid	C_30_H_48_O_3_	Lipids and lipid-like molecules	3.210	1.72E^-07^	1.713
LPS 15:0	C_21_H_42_NO_9_P	Lipids and lipid-like molecules	5.723	3.36E^-07^	1.830
Glycyl-L-leucine	C_8_H_16_N_2_O_3_	Organic acids and derivatives	1.279	3.59E^-07^	1.774
ST 28:1;O;Hex;FA 18:2	C_52_H_88_O_7_	Lipids and lipid-like molecules	2.559	3.86E^-07^	1.333
Octadec-9-ynoic acid	C_18_H_32_O_2_	Organic acids and derivatives	3.265	4.69E^-07^	1.718
Oleanolic acid	C_30_H_48_O_3_	Organic acids and derivatives	2.811	5.30E^-07^	1.749
PE 18:0§§	C_23_H_46_NO_8_P	Lipids and lipid-like molecules	3.876	5.58E^-07^	1.687
15-OxoEDE	C_20_H_34_O_3_	Lipids and lipid-like molecules	6.576	6.57E^-07^	1.746
ERH||	C_17_H_28_N_8_O_6_	Organic nitrogen compounds	2.566	6.82E^-07^	1.319
Lysopc 18:2	C_26_H_50_NO_7_P	Lipids and lipid-like molecules	3.318	7.01E^-07^	1.798
FAHFA 19:2/18:2	C_37_H_64_O_4_	Lipids and lipid-like molecules	4.476	7.49E^-07^	1.839

Note: †log2FC: log2FoldChange; ‡VIP: Variable importance in projection; §LPC: Lysophosphatidylcholine; |MGDG: Monogalactosyldiacylglycerol; ¶FAHFAs: fatty acid esters of hydroxyl fatty acids; #HKK: His-Lys-Lys; ††LPE: Lysophosphatidyl ethanolamine; ‡‡LPS: Lipopolysaccharides; §§PE 18:0: 1-stearoyl-2-oleoyl-sn-glycero-3-phosphoethanolamine; ||ERH: Erythrohydrobupropion.

## ﻿Discussion

This study provides insights into the genomic characteristics and potential evolutionary adaptations of *C.blackwelliae*. The genome size of 31.06 Mb, within the established range of available *Cordyceps* genomes (30.10–34.97 Mb) (Suppl. material 1: table S1), suggests a conserved genome size across the genus. However, the lower number of protein-coding genes (8,138) compared to other *Cordyceps* species/strains (8,705–11,441) (Suppl. material 1: table S1) may indicate a streamlined genome, potentially reflecting ecological specialisation or gene loss due to evolutionary pressure. Additionally, the significantly greater average length of protein-coding genes in *C.blackwelliae* (2,256.47 bp) compared to *C.militaris* and *C.pseudotenuipes* (1,527–1,742 bp) suggests differences in gene structure (Suppl. material 1: table S1), possibly involving more complex regulatory regions, additional functional domains or alternative splicing mechanisms. The shared enrichment of COG categories, such as “Carbohydrate transport and metabolism”, “Amino acid transport and metabolism” and “Translation, ribosomal structure and biogenesis” with *C.pseudotenuipes*, suggests conserved metabolic and biosynthetic pathways essential for *Cordyceps* growth and development (Lu et al. 2022). In addition, the unique enrichment of *C.blackwelliae* genes in “Inorganic ion transport and metabolism” and “Lipid transport and metabolism” may indicate specialised adaptations in nutrient acquisition, warranting further ecological and functional studies.

Genomic analyses indicated that the *MAT1-2* mating type locus in *C.blackwelliae* comprises the complete *MAT1-2-1* gene and a truncated version of *MAT1-1-1*, which has lost its N-terminal α-box domain, a pattern also reported in *C.chanhua* (Lu et al. 2017) and observed across all currently available *Cordyceps* genomes (Fig. 1a). This truncation may result from recombination or crossover event, a phenomenon similarly documented in non-*Cordyceps* fungi, such as *Ophiostomamontium* (Tsui et al. 2013), suggesting a potentially widespread occurrence of *MAT* gene truncation. Expression analysis showed higher transcription level of *MAT1-2-1* during the mycelial growth stages (i.e. LM and WM stages) than during fruiting body development (i.e. FB stage). The truncated *MAT1-1-1* of *C.blackwelliae* showed extremely low expression under all examined conditions, a pattern also reported in *C.chanhua* (Lu et al. 2017).

Comparative transcriptome analysis revealed stage-specific gene expression patterns associated with fungal development. At the LM stage, genes associated with DNA repair and recombination, as well as membrane component were highly expressed, supporting rapid cell division, biomass accumulation and adaptation to the liquid environment. The WM stage was characterised by the up-regulation of genes related to enzyme activity and co-factor binding, enhancing mycelial nutrient acquisition and supporting robust growth on solid substrates. During the FB stage, genes involved in various biosynthetic and catabolic processes were significantly overexpressed, facilitating morphological and physiological transitions required for fruiting body development. These findings align with transcriptomic studies on other *Cordyceps* species (Zheng et al. 2011; Yin et al. 2012).

Key regulatory genes identified at each developmental stage further highlight functional specialisation. The hub genes at the LM stage were closely associated with key phenotypic traits, including rapid growth, branching, nutrient utilisation and stress adaptation. For example, the *ARP3* (*gene 3273*) gene promotes hyphal apical formation and non-polar growth in *Neurospora* (Tinsley et al. 1998), suggesting that vegetative growth is the primary process at the LM stage. *HXT1* (*gene 1654*) enhances glucose uptake, supporting efficient nutrient utilisation and biomass accumulation (Reifenberger et al. 1997). CIR2 (*gene0726*) encoding a putative electron transfer flavoprotein-ubiquinone oxidoreductase, generates ATP via electron transfer to boost biomass and accelerate growth cycles in liquid media (Watmough et al. 2010), while its mitochondrial dynamics ensure linear hyphal extension by delivering localised ATP to growing tips (Bereiter-Hahn et al. 1994). *LAC1* (*gene 4456*) contributes to oxidative stress tolerance, enabling mycelia to thrive in liquid environments (Baldrian 2006). The identified hub genes at the WM stage drive key phenotypic adaptations for mycelial colonisation on solid wheat substrates. For instance, *AOX1* (*gene 0971*) may mitigate oxidative stress and modulate energy metabolism in the solid environment (Dunn 2023), while *SSN2* (*gene 1618*) could transduce surface or nutrient signals to guide hyphal growth patterns (Hengartner et al. 1998). *CHS7* (*gene 6243*)-mediated chitin deposition likely reinforces cell wall integrity, preventing lysis during hyphal penetration of wheat tissues (Lenardon et al. 2010). Collectively, these genetic programmes likely underpin phenotypic hallmarks of solid-culture mycelia, including increased adherence, directional growth and structural resilience compared to liquid-grown counterparts. Additionally, ribosomal genes are up-regulated at the WM stage (Suppl. material 2: fig. S5a), corroborating findings that such genes are highly expressed at early developmental stages of most species, followed by a gradual decline in expression in both young and mature fruiting bodies (Shu et al. 2022; Nagy et al. 2023). This expression pattern likely reflects the substantial protein requirements of rapidly growing tissues at the WM stage and supports a biphasic model of fruiting body development, characterised by distinct differentiation and growth phases (Nagy et al. 2023). Moreover, cell wall remodelling has been established as an essential process during fruiting body development. The cell wall must preserve structural integrity and defence mechanisms while providing sufficient plasticity to accommodate morphological changes throughout development. This dynamic process is crucial for the phenotypic traits, such as size, shape and robustness of fruiting bodies. Chitin synthases, chitin deacetylases and glucanases are primarily responsible for this dynamic rearrangement (Nagy et al. 2023). In *C.blackwelliae*, genes encoding chitin deacetylase and β-1,3-glucanase were up-regulated at the FB stage (Suppl. material 2: fig. S5b), consistent with previous reports indicating that these enzymes are highly expressed in the elongation region of fruiting bodies (Wang et al. 2018; Patel et al. 2019; Bai et al. 2020). Furthermore, the septin protein family, which is implicated in cell morphogenesis, development and production, exhibited significant overexpression during the FB stage (Suppl. material 2: fig. S5c) (Gladfelter 2006). This protein has been identified as a key gene for fruiting body elongation in *Coprinopsiscinerea* and *Agaricusbisporus* (Lu et al. 2018; Shioya et al. 2013).

Metabolomic analysis identified 1,161 metabolites across three distinct developmental stages of *C.blackwelliae*. These metabolites were primarily categorised as lipids, organic acids, amino acids, nucleosides, carbohydrates, vitamins and alkaloids, aligning with previous metabolomic studies on *Cordyceps* (Liu et al. 2021; Guo et al. 2022; Wang et al. 2023). The observed variations in metabolite abundance during development offer valuable insights into the metabolic transitions and regulatory mechanisms that govern fruiting body development. At the WM stage, metabolic activity appears to be directed towards the accumulation of precursors and the establishment of a conducive cellular environment, as evidenced by an increase in unsaturated fatty acid levels (Suppl. material 2: fig. S15c). The accumulation of unsaturated fatty acids is crucial for the formation of cell membranes with optimal fluidity and permeability (Yang et al. 2011), which is essential for the remodelling of the cell wall necessary for subsequent fruiting body development. As development progresses towards the FB stage, there is a notable reprogramming of metabolic pathways to accommodate the increased demands for energy, biosynthesis and morphogenesis. At this stage, there is a marked increase in the levels of essential amino acids, including valine, L-lysine, threonine, tryptophan and L-phenylalanine (Suppl. material 2: fig. S13), indicating their active participation in protein synthesis for the formation of new tissues and structures of fruiting bodies (Liu et al. 2024). This observation aligns with the elevated concentrations of amino acids reported in *Ophiocordycepsgracilis* at the fruiting body stage (Wang et al. 2023). In terms of carbohydrate metabolism, the degradation of stored polysaccharides was noted (Suppl. material 2: fig. S15b), which was accompanied by an increase in the levels of ATP and other high-energy phosphate compounds, suggesting enhanced energy production to support the energy-intensive processes of cell division, elongation and the biosynthesis of complex macromolecules, which are critical for the construction of the fruiting body’s cell walls.

A conjoint transcriptomic and metabolomic analysis identified 17 genes and 34 metabolites likely involved in fruiting body development. Amongst these genes, *gene 0752*, which encodes a putative DSBA-like thioredoxin domain protein, is associated with redox regulation (Nilewski et al. 2021) and may play a pivotal role in safeguarding developing structures from oxidative damage, thereby ensuring proper morphogenesis (Zhang et al. 2021). *Gene 2568*, which encodes putative mannosyl-oligosaccharide 1,2-alpha-mannosidase IB, is involved in N-glycan processing (Vallée et al. 2000). This processing is vital for the secretion and functionality of proteins that drive fruiting body development (Buser et al. 2010). LPQL (*gene 3092*), which corresponds to lipoprotein aminopeptidase, acts on lipoprotein-associated peptides (Cahan et al. 2001) and may impact membrane properties and intercellular communication during fruiting body formation (Melly et al. 2019). *Gene 4853*, a putative regulator of G protein-signalling superfamily member, governs G protein-coupled receptor signalling, which is essential for transducing environmental signals that can trigger developmental changes, including the initiation of fruiting body development (Cabrera et al. 2022). *Genes 5670* (chitin deacetylase) and *6604* (exo-beta-D-glucosaminidase) are involved in the degradation of chitin and chitosan, processes that may be critical for cell wall remodelling during fruiting body development (Nagy et al. 2023). *Gene 6756*, which encodes putative mannose-binding lectin, may facilitate cell recognition and adhesion processes by binding to mannose-containing glycoconjugates and is a significant component of the innate immune system (Hammad et al. 2018), potentially influencing the interactions between the fungus and its environment. Furthermore, indoles and tryptophan, two of the 34 metabolites identified by correlation analysis, have been proved to affect fruiting body induction via cAMP signalling (Sakamoto 2018). These findings offer valuable insights into the genetic regulation of fruiting body formation in *C.blackwelliae*.

## ﻿Conclusion

This study provides the first reference genome for *C.blackwelliae*, laying a foundation for future functional studies. Integrated transcriptomic and metabolomic analyses identified 17 genes and 34 metabolites likely involved in fruiting body development. However, their precise roles in *C.blackwelliae*’s life cycle remain to be validated. Future research should focus on elucidating the specific functions of these genes and metabolites in fungal growth and development.

## ﻿Additional information

### Adherence to national and international regulations

All the fungal strains used in this study have been legally obtained, respecting the Convention on Biological Diversity (Rio Convention).

### Author ORCIDs

Shu Zhang https://orcid.org/0009-0009-3779-1700

Yong-Jie Zhang https://orcid.org/0000-0002-6331-2189

### Data availability

The genome assembly of *Cordycepsblackwelliae* ZYJ0835 has been deposited at NCBI with an accession no. JBKZWQ000000000. RNA-seq data have been deposited at NCBI with a BioProject no. PRJNA1214394.

## Supplementary materials

Supplementary material 1 Supplementary tables S1–S16 Data type xlsx Explanation note **table S1.** Genome information of *Cordyceps* species/strains with available whole genome sequences. **table S2.***Cordyceps* species/strain and corresponding scaffolds harbouring *MAT* genes. **table S3.** Fungal taxa used in nuclear phylogenomic analyses. **table S4.** Fungal taxa used in mitochondrial phylogenetic analyses. **table S5.** Primers used in RT-qPCR assays. **table S6**. Statistics for scaffold length and telomere repeats in the genome assembly of *Cordycepsblackwelliae*. **table S7.** Summary of annotations for the *Cordycepsblackwelliae* genome. **table S8.** Transcriptional expression of *MAT1-2-1* and the truncated *MAT1-1-1* in *Cordycepsblackwelliae*. **table S9.** Summary of sequencing data of the *Cordycepsblackwelliae* transcriptome and mapping rates. **table S10.** List of novel genes identified by RNA-seq. **table S11.**DEGs across three groups of samples when |log2FoldChange| > 2. **table S12.** Description of extremely significant DEGs in volcano plot. **table S13.**GO and KEGG enrichment analysis of DEGs. **table S14.** Genes with developmental stage-specific alternative splicing. **table S15.** Genes of turquoise, magenta and black modules and hub genes. **table S16.** Statistics of metabolites present in *Cordycepsblackwelliae*. https://binary.pensoft.net/file/1323860This dataset is made available under the Open Database License (http://opendatacommons.org/licenses/odbl/1.0/). The Open Database License (ODbL) is a license agreement intended to allow users to freely share, modify, and use this Dataset while maintaining this same freedom for others, provided that the original source and author(s) are credited.Jia-Ni Li, Shu Zhang, Yong-Jie Zhang

Supplementary material 2 Supplementary figures S1–S16 Data type docx Explanation note **figure S1.** Soft threshold determination of WGCNA. **figure S2.** Statistics of functional annotations for the *Cordycepsblackwelliae* genome. **figure S3.** Functional annotation of the *Cordycepsblackwelliae* genome. **figure S4.** Principal component analysis (PCA) and Venn diagram of DEGs in RNA-seq. **figure S5.** Expression heatmap of development related genes in *Cordycepsblackwelliae*. **figure S6.** Validation of RNA-seq results by RT-qPCR analyses. **figure S7.** Detailed sashimi plots of genes exhibiting developmental stage-specific alternative splicing (FRD < 0.05). **figure S8.**GO term enrichment analysis for genes undergoing alternative splicing. **figure S9.** The total ion chromatogram of three stages in both positive (a) and negative (b) ion modes. **figure S10.** Functional annotation of Cordycepsblackwelliae metabolites. **figure S11.** Multivariate statistics in three comparisons. **figure S12.** Venn diagram of DMs by pairwise comparison. **figure S13.** Heatmap depicting the differential metabolic profile of amino acids. **figure S14.** Relative content of corey lactone diol across three developmental stages. **figure S15.** Interactive pathway analysis of *Cordycepsblackwelliae* in the FB vs. WM comparison. **figure S16.** Heatmap of 34 metabolites positively correlated with Group1 genes in three stages. https://binary.pensoft.net/file/1323861This dataset is made available under the Open Database License (http://opendatacommons.org/licenses/odbl/1.0/). The Open Database License (ODbL) is a license agreement intended to allow users to freely share, modify, and use this Dataset while maintaining this same freedom for others, provided that the original source and author(s) are credited.Jia-Ni Li, Shu Zhang, Yong-Jie Zhang

## References

[B1] Ashburner M Ball CA (2000) Gene ontology: tool for the unification of biology. Nature Genetics 25(1): 25–29. 10.1038/75556 10802651 PMC3037419

[B2] Bai Y Wang Y-X (2020) Heterologous expression and characterization of a novel chitin deacetylase, *CDA3*, from the mushroom *Coprinopsiscinerea*. International Journal of Biological Macromolecules 150: 536–545. 10.1016/j.ijbiomac.2020.02.083 32057882

[B3] Baldrian P (2006) Fungal laccases - occurrence and properties. FEMS Microbiology Reviews 30(2): 215–242. 10.1111/j.1574-4976.2005.00010.x 16472305

[B4] Benesty J Chen J (2009) Pearson Correlation Coefficient. In: Noise Reduction in Speech Processing. Springer, Berlin, Heidelberg, 37–40. 10.1007/978-3-642-00296-0_5

[B5] Benjamini Y Hochberg Y (2018) Controlling the false discovery rate: a practical and powerful approach to multiple testing. The Journal of the Royal Statistical Society, Series B (Statistical Methodology) 57(1): 289–300. 10.1111/j.2517-6161.1995.tb02031.x

[B6] Bereiter-Hahn J Vöth M (1994) Dynamics of mitochondria in living cells: shape changes, dislocations, fusion, and fission of mitochondria. Microscopy Research and Technique 27(3): 198–219. 10.1002/jemt.1070270303 8204911

[B7] Blin K Shaw S (2023) antiSMASH 7.0: new and improved predictions for detection, regulation, chemical structures and visualisation. Nucleic Acids Research 51: w46–w50. 10.1093/nar/gkad344 PMC10320115 37140036

[B8] Buchfink B Reuter K Drost HG (2021) Sensitive protein alignments at tree-of-life scale using diamond. Nature Methods 18(4): 366–368. 10.1038/s41592-021-01101-x 33828273 PMC8026399

[B9] Buser R Lazar Z (2010) Identification, characterization, and biosynthesis of a novel N-glycan modification in the fruiting body of the basidiomycete *Coprinopsiscinerea*. Journal of Biological Chemistry 285(14): 10715–10723. 10.1074/jbc.M109.076075 20061575 PMC2856279

[B10] Cabrera IE Oza Y (2022) Regulator of G protein signaling proteins control growth, development and cellulase production in *Neurosporacrassa*. Journal of Fungi 8(10): 1076. 10.3390/jof8101076 36294641 PMC9604755

[B11] Cahan R Axelrad I (2001) A secreted aminopeptidase of *Pseudomonasaeruginosa*, identification, primary structure, and relationship to other aminopeptidases. Journal of Biological Chemistry 276(47): 43645–43652. 10.1074/jbc.M106950200 11533066

[B12] Capella-Gutiérrez S Silla-Martínez JM Gabaldón T (2009) trimAI: a tool for automated alignment trimming in large-scale phylogenetic analyses. Bioinformatics 25(15): 1972–1973. 10.1093/bioinformatics/btp348 19505945 PMC2712344

[B13] Chai L-S Li J-M (2024) Genomic and transcriptome analysis reveals the biosynthesis network of cordycepin in *Cordycepsmilitaris*. Genes 15(5): 626. 10.3390/genes15050626 38790255 PMC11120935

[B14] Chen B Sun Y-L (2020) Bioactive metabolites and potential mycotoxins produced by *Cordyceps* fungi: a review of safety. Toxins 12(6): 410. 10.3390/toxins12060410 32575649 PMC7354514

[B15] Chen G Shi T-L Shi L-M (2017) Characterizing and annotating the genome using RNA-seq data. Science China Life Sciences 60(2): 116–125. 10.1007/s11427-015-0349-4 27294835

[B16] Chen S-F Zhou Y-Q (2018) fastp: an ultra-fast all-in-one FASTQ preprocessor. Bioinformatics 34(17): i884–i890. 10.1093/bioinformatics/bty560 PMC6129281 30423086

[B17] Dong C-H Guo S-P (2015) *Cordyceps* industry in China. Mycology 6(2): 121–129. 10.1080/21501203.2015.1043967 30151320 PMC6106062

[B18] Duan D-E (2019) Taxonomy and phylogeny of *Cordycipitaceae* from Vietnam. Master’s Thesis, Yunnan University, Kunming, Yunnan.

[B19] Duan M-Z Long S-F (2023) Genome, transcriptome, and metabolome analyses provide new insights into the resource development in an edible fungus *Dictyophoraindusiata*. Frontiers in Microbiology 14: 1137159. 10.3389/fmicb.2023.1137159 PMC9948255 36846778

[B20] Dunn AK (2023) Alternative oxidase in bacteria. Biochimica et Biophysica Acta 1864(1): 148929. 10.1016/j.bbabio.2022.148929 36265564

[B21] Eddy SR (2011) Accelerated profile HMM searches. PLOS Computational Biology 7(10): e1002195. 10.1371/journal.pcbi.1002195 PMC3197634 22039361

[B22] Edgar RC (2004) Muscle: a multiple sequence alignment method with reduced time and space complexity. BMC Bioinformatics 5: 113. 10.1186/1471-2105-5-113 PMC517706 15318951

[B23] Fan X-P Zhang S Zhang Y-J (2022) Evaluation of biological activities and artificial cultivation of fruiting bodies of *Cordycepsblackwelliae*. Mycosystema 41(11): 1807–1818.

[B24] Finn RD Coggill P (2016) The Pfam protein families database: towards a more sustainable future. Nucleic Acids Research 44(D1): D279–D285. 10.1093/nar/gkv1344 PMC4702930 26673716

[B25] Gladfelter AS (2006) Control of filamentous fungal cell shape by septins and formins. Nature Reviews Microbiology 4(3): 223–229. 10.1038/nrmicro1345 16429163

[B26] Götz S García-Gómez JM (2008) High-throughput functional annotation and data mining with the Blast2Go suite. Nucleic Acids Research 36(10): 3420–3435. 10.1093/nar/gkn176 18445632 PMC2425479

[B27] Guo S Lin M-T (2022) Comparative metabolic profiling of wild *Cordyceps* species and their substituents by liquid chromatography-tandem mass spectrometry. Frontiers in Pharmacology 13: 1036589. 10.3389/fphar.2022.1036589 PMC9729555 36506548

[B28] Hammad NM El Badawy NE (2018) Mannose-binding lectin: a potential therapeutic candidate against *Candida* infection. BioMed Research International 2018: 2813737. 10.1155/2018/2813737 PMC5954966 29854737

[B29] He L Xie F (2023) Transcriptome and metabonomics combined analysis revealed the energy supply mechanism involved in fruiting body initiation in Chinese cordyceps. Scientific Reports 13(1): 9500. 10.1038/s41598-023-36261-7 37308669 PMC10261108

[B30] Hengartner CJ Myer VE (1998) Temporal regulation of RNA polymerase II by SRB10 and KIN28 cyclin-dependent kinases. Molecular Cell 2(1): 43–53. 10.1016/S1097-2765(00)80112-4 9702190

[B31] Holt C Yandell M (2011) Maker2: an annotation pipeline and genome-database management tool for second-generation genome projects. BMC Bioinformatics 12: 491. 10.1186/1471-2105-12-491 PMC3280279 22192575

[B32] Kanehisa M Goto S (2000) KEGG: kyoto encyclopedia of genes and genomes. Nucleic Acids Research 28(1): 27–30. 10.1093/nar/28.1.27 10592173 PMC102409

[B33] Kim D Langmead B Salzberg SL (2015) HISAT: a fast spliced aligner with low memory requirements. Nature Methods 12(4): 357–360. 10.1038/nmeth.3317 25751142 PMC4655817

[B34] Koren S Walenz BP (2017) Canu: scalable and accurate long-read assembly via adaptive k-mer weighting and repeat separation. Genome Research 27(5): 722–736. 10.1101/gr.215087.116 28298431 PMC5411767

[B35] Korf I (2004) Gene finding in novel genomes. BMC Bioinformatics 5: 59. 10.1186/1471-2105-5-59 PMC421630 15144565

[B36] Krogh A Larsson B (2001) Predicting transmembrane protein topology with a hidden markov model: application to complete genomes. Journal of Molecular Biology 305(3): 567–580. 10.1006/jmbi.2000.4315 11152613

[B37] Langfelder P Horvath S (2008) WGCNA: an R package for weighted correlation network analysis. BMC Bioinformatics 9: 559. 10.1186/1471-2105-9-559 PMC2631488 19114008

[B38] Lee RC Farfan-Caceres L (2021) Reference genome assembly for Australian *Ascochytalentis* isolate Al4. G3 11(2): jkab006. 10.1093/g3journal/jkab006 PMC8022934 33604672

[B39] Lenardon MD Munro CA Gow NA (2010) Chitin synthesis and fungal pathogenesis. Current Opinion in Microbiology 13(4): 416–423. 10.1016/j.mib.2010.05.002 20561815 PMC2923753

[B40] Lei R Duan W-J (2023) Nanopore/illumina hybrid whole-genome sequence resource of *Plenodomuslindquistii* strain US01 infecting sunflower. Plant Disease 107(6): 1929–1933. 10.1094/PDIS-09-22-2055-A 36510424

[B41] Li J-N Zhang S Zhang Y-J (2025a) Optimization of mycelial culture conditions of the entomopathogenic fungus *Cordycepsblackwelliae* in submerged culture. Mycological Progress 24(1): 10. 10.1007/s11557-024-02028-1

[B42] Li J-N Zhang S Zhang Y-J (2025b) Screening of reference genes for real-time quantitative reverse transcription PCR in *Cordycepsblackwelliae*. Microbiology China 52(1): 219–229.

[B43] Li X Liu Q-P (2019) A breakthrough in the artificial cultivation of Chinese cordyceps on a large-scale and its impact on science, the economy, and industry. Critical Reviews in Biotechnology 39(2): 181–191. 10.1080/07388551.2018.1531820 30394122

[B44] Liao Y Smyth GK Shi W (2014) Featurecounts: an efficient general purpose program for assigning sequence reads to genomic features. Bioinformatics 30(7): 923–930. 10.1093/bioinformatics/btt656 24227677

[B45] Liu Q Huang B (2024) Analysis of differential metabolites during fruiting body development of *Pleurotusostreatus* based on untargeted metabolomics. China Cucurbits and Vegetables 37(01): 45–55.

[B46] Liu W-Z Xie Y-B (2015) IBS: an illustrator for the presentation and visualization of biological sequences. Bioinformatics 31(20): 3359–3361. 10.1093/bioinformatics/btv362 26069263 PMC4595897

[B47] Liu Y-F Xiao K (2021) Comparison of metabolism substances in *Cordycepssinensis* and *Cordycepsmilitaris* cultivated with tussah pupa based on LC-MS. Journal of Food Biochemistry 45(6): e13735. 10.1111/jfbc.13735 33890309

[B48] Livak KJ Schmittgen TD (2001) Analysis of relative gene expression data using real-time quantitative PCR and the 2(-delta delta C(T)) method. Methods 25(4): 402–408. 10.1006/meth.2001.1262 11846609

[B49] Love MI Huber W Anders S (2014) Moderated estimation of fold change and dispersion for RNA-seq data with DESeq2. Genome Biology 15(12): 550. 10.1186/s13059-014-0550-8 25516281 PMC4302049

[B50] Lowe TM Eddy SR (1997) tRNAscan-SE: a program for improved detection of transfer RNA genes in genomic sequence. Nucleic Acids Research 25(5): 955–964. 10.1093/nar/25.5.955 9023104 PMC146525

[B51] Lu Y-L Wang Y (2022) Genomic comparative analysis of *Cordycepspseudotenuipes* with other species from *Cordyceps*. Metabolites 12(9): 844. 10.3390/metabo12090844 36144248 PMC9505148

[B52] Lu Y-P Guo Z-J (2018) Cloning and expression analyses of the septin gene *Ab.Cdc3* in *Agaricusbisporus*. Mycosystema 37(12): 1635–1642.

[B53] Lu Y-Z Luo F-F (2017) Omics data reveal the unusual asexual-fruiting nature and secondary metabolic potentials of the medicinal fungus *Cordycepscicadae*. BMC Genomics 18(1): 668. 10.1186/s12864-017-4060-4 28854898 PMC5577849

[B54] Lukashin AV Borodovsky M (1998) GeneMark.hmm: new solutions for gene finding. Nucleic Acids Research 26(4): 1107–1115. 10.1093/nar/26.4.1107 9461475 PMC147337

[B55] Melly GC Stokas H (2019) Structural and functional evidence that lipoprotein LpqN supports cell envelope biogenesis in *Mycobacteriumtuberculosis*. Journal of Biological Chemistry 294(43): 15711–15723. 10.1074/jbc.RA119.008781 31471317 PMC6816100

[B56] Mongkolsamrit S Noisripoom W (2018) Disentangling cryptic species with isaria-like morphs in *Cordycipitaceae*. Mycologia 110(1): 230–257. 10.1080/00275514.2018.1446651 29863995

[B57] Nagy LG Vonk PJ (2023) Lessons on fruiting body morphogenesis from genomes and transcriptomes of agaricomycetes. Studies in Mycology 104: 1–85. 10.3114/sim.2022.104.01 37351542 PMC10282164

[B58] Nguyen LT Schmidt HA (2015) IQ-TREE: a fast and effective stochastic algorithm for estimating maximum-likelihood phylogenies. Molecular Biology and Evolution 32(1): 268–274. 10.1093/molbev/msu300 25371430 PMC4271533

[B59] Nilewski S Varatnitskaya M (2021) Functional metagenomics of the thioredoxin superfamily. Journal of Biological Chemistry 296: 100247. 10.1074/jbc.RA120.016350 PMC7949104 33361108

[B60] Parra G Bradnam K Korf I (2007) CEGMA: a pipeline to accurately annotate core genes in eukaryotic genomes. Bioinformatics 23(9): 1061–1067. 10.1093/bioinformatics/btm071 17332020

[B61] Patel PK Free SJ (2019) The genetics and biochemistry of cell wall structure and synthesis in *Neurosporacrassa*, a model filamentous fungus. Frontiers in Microbiology 10: 2294. 10.3389/fmicb.2019.02294 PMC6796803 31649638

[B62] Pertea M Pertea GM (2015) StringTie enables improved reconstruction of a transcriptome from RNA-seq reads. Nature Biotechnology 33(3): 290–295. 10.1038/nbt.3122 PMC4643835 25690850

[B63] Petersen TN Brunak S (2011) SignalP 4.0: discriminating signal peptides from transmembrane regions. Nature Methods 8(10): 785–786. 10.1038/nmeth.1701 21959131

[B64] Qu Q-S Yang F (2019) Analysis of the bacteria community in wild *Cordycepscicadae* and its influence on the production of hea and nucleosides in *Cordycepscicadae*. Journal of Applied Microbiology 127(6): 1759–1767. 10.1111/jam.14432 31463998

[B65] Rambaut A (2009) FigTree, a graphical viewer of phylogenetic trees. PLOS Computational Biology 5: 9–10.

[B66] Reifenberger E Boles E Ciriacy M (1997) Kinetic characterization of individual hexose transporters of saccharomyces cerevisiae and their relation to the triggering mechanisms of glucose repression. European Journal of Biochemistry 245(2): 324–333. 10.1111/j.1432-1033.1997.00324.x 9151960

[B67] Rohart F Gautier B (2017) Mixomics: an R package for ‘omics feature selection and multiple data integration. PLOS Computational Biology 13(11): e1005752. 10.1371/journal.pcbi.1005752 PMC5687754 29099853

[B68] Sakamoto Y (2018) Influences of environmental factors on fruiting body induction, development and maturation in mushroom-forming fungi. Fungal Biology Reviews 32: 1749–4613. 10.1016/j.fbr.2018.02.003

[B69] Sayers EW Bolton EE (2022) Database resources of the national center for biotechnology information. Nucleic Acids Research 50(D1): D20–D26. 10.1093/nar/gkab1112 PMC8728269 34850941

[B70] Schultz J Milpetz F (1998) SMART, a simple modular architecture research tool: identification of signaling domains. Proceedings of the National Academy of Sciences of the United States of America 95(11): 5857–5864. 10.1073/pnas.95.11.5857 9600884 PMC34487

[B71] Schimmel TG Coffman AD Parsons SJ (1998) Effect of butyrolactone I on the producing fungus, *Aspergillusterreus*. Applied and Environmental Microbiology 64(10): 3707–3712. 10.1128/AEM.64.10.3707-3712.1998 9758788 PMC106526

[B72] Seppey M Manni M Zdobnov EM (2019) BUSCO: assessing genome assembly and annotation completeness. Methods in Molecular Biology 1962: 227–245. 10.1007/978-1-4939-9173-0_14 31020564

[B73] Shannon P Markiel A (2003) Cytoscape: a software environment for integrated models of biomolecular interaction networks. Genome Research 13(11): 2498–2504. 10.1101/gr.1239303 14597658 PMC403769

[B74] Shen N Xie H-Y (2024) Near-gapless genome and transcriptome analyses provide insights into fruiting body development in *Lentinulaedodes*. International Journal of Biological Macromolecules 263(Pt 2): 130610. 10.1016/j.ijbiomac.2024.130610 38447851

[B75] Shen S Park JW (2014) rMATS: robust and flexible detection of differential alternative splicing from replicate RNA-seq data. Proceedings of the National Academy of Sciences of the United States of America 111(51): E5593–5601. 10.1073/pnas.1419161111 PMC4280593 25480548

[B76] Shioya T Nakamura H (2013) The *Coprinopsiscinerea* septin *Cc.Cdc3* is involved in stipe cell elongation. Fungal Genetics and Biology 58–59: 80–90. 10.1016/j.fgb.2013.08.007 23973959

[B77] Shu L-L Wang M-Y (2022) De novo transcriptome assembly and comprehensive assessment provide insight into fruiting body formation of *Sparassislatifolia*. Scientific Reports 12(1): 11075. 10.1038/s41598-022-15382-5 35773379 PMC9247108

[B78] Simão FA Waterhouse RM (2015) BUSCO: assessing genome assembly and annotation completeness with single-copy orthologs. Bioinformatics 31(19): 3210–3212. 10.1093/bioinformatics/btv351 26059717

[B79] Stanke M Keller O (2006) AUGUSTUS: ab initio prediction of alternative transcripts. Nucleic Acids Research 34: W435–W439. 10.1093/nar/gkl200 PMC1538822 16845043

[B80] Tamura K Stecher G Kumar S (2021) MEGA11: molecular evolutionary genetics analysis version 11. Molecular Biology and Evolution 38(7): 3022–3027. 10.1093/molbev/msab120 33892491 PMC8233496

[B81] Tatusov RL Galperin MY (2000) The COG database: a tool for genome-scale analysis of protein functions and evolution. Nucleic Acids Research 28(1): 33–36. 10.1093/nar/28.1.33 10592175 PMC102395

[B82] Tinsley JH Lee IH (1998) Analysis of actin and actin-related protein 3 (*ARP3*) gene expression following induction of hyphal tip formation and apolar growth in *Neurospora*. Molecular Genetics and Genomics 259(6): 601–609. 10.1007/s004380050853 9819052

[B83] Tsui CK DiGuistini S (2013) Unequal recombination and evolution of the mating-type (*MAT*) loci in the pathogenic fungus *Grosmanniaclavigera* and relatives. G3 3(3): 465–480. 10.1534/g3.112.004986 PMC3583454 23450093

[B84] Vallée F Lipari F (2000) Crystal structure of a class I alpha1,2-mannosidase involved in N-glycan processing and endoplasmic reticulum quality control. The EMBO Journal 19(4): 581–588. 10.1093/emboj/19.4.581 10675327 PMC305596

[B85] Walker BJ Abeel T (2014) Pilon: an integrated tool for comprehensive microbial variant detection and genome assembly improvement. PLOS ONE 9(11): e112963. 10.1371/journal.pone.0112963 PMC4237348 25409509

[B86] Wang Y Yang L-H (2023) Comparative metabolic profiling of mycelia, fermentation broth, spore powder and fruiting bodies of *Ophiocordycepsgracilis* by LC-MS/MS. Phytochemical Analysis 34(8): 984–996. 10.1002/pca.3266 37482969

[B87] Wang Y-X Niu X (2018) Heterologous expression, characterization and possible functions of the chitin deacetylases, *Cda1* and *Cda2*, from mushroom *Coprinopsiscinerea*. Glycobiology 28(5): 318–332. 10.1093/glycob/cwy007 29370398

[B88] Watmough NJ Frerman FE (2010) The electron transfer flavoprotein: ubiquinone oxidoreductases. Biochimica et Biophysica Acta 1797(12): 1910–1916. 10.1016/j.bbabio.2010.10.007 20937244

[B89] Wei T Simko V (2017) Corrplot: visualization of a correlation matrix. Journal of Statistical Software 48(1): 1–27.

[B90] Wei Y-Q Zhang L (2021) Chinese caterpillar fungus (*Ophiocordycepssinensis*) in China: current distribution, trading, and futures under climate change and overexploitation. Science of the Total Environment 755(Pt 1): 142548. 10.1016/j.scitotenv.2020.142548 PMC7521209 33035977

[B91] Wen B Mei Z-L (2017) metaX: a flexible and comprehensive software for processing metabolomics data. BMC Bioinformatics 18(1): 183. 10.1186/s12859-017-1579-y 28327092 PMC5361702

[B92] Westerhuis JA van Velzen EJ (2010) Multivariate paired data analysis: multilevel PLSDA versus OPLSDA. Metabolomics 6(1): 119–128. 10.1007/s11306-009-0185-z 20339442 PMC2834771

[B93] Wickham H (2009) ggplot2: elegant graphics for data analysis. Springer, New York, 202 pp. 10.1007/978-0-387-98141-3

[B94] Wu X Du Z-H (2024) Integrative analysis of transcriptome and metabolome sheds light on flavonoid biosynthesis in the fruiting body of *Strophariarugosoannulata*. Journal of Fungi 10(4): 254. 10.3390/jof10040254 38667925 PMC11051051

[B95] Yang X-G Sheng W-W (2011) Effects of fatty acid unsaturation numbers on membrane fluidity and α-secretase-dependent amyloid precursor protein processing. Neurochemistry International 58(3): 321–329. 10.1016/j.neuint.2010.12.004 21184792 PMC3040984

[B96] Yin Y-L Yu G-J (2012) Genome-wide transcriptome and proteome analysis on different developmental stages of *Cordycepsmilitaris*. PLoS ONE 7(12): e51853. 10.1371/journal.pone.0051853 PMC3522581 23251642

[B97] Yu G Wang L-G (2012) Clusterprofiler: an R package for comparing biological themes among gene clusters. Omics 16(5): 284–287. 10.1089/omi.2011.0118 22455463 PMC3339379

[B98] Zhang H Yue P (2021) mRNA-seq and miRNA-seq profiling analyses reveal molecular mechanisms regulating induction of fruiting body in *Ophiocordycepssinensis*. Scientific Reports 11(1): 12944. 10.1038/s41598-021-91718-x 34155233 PMC8217512

[B99] Zhang Y-J Zhang S (2010) A simple method of genomic DNA extraction suitable for analysis of bulk fungal strains. Letters in Applied Microbiology 51(1): 114–118. 10.1111/j.1472-765X.2010.02867.x 20536704

[B100] Zhang Y-J Fan X-P (2023) Mitochondrial genome of *Cordycepsblackwelliae*: organization, transcription, and evolutionary insights into *Cordyceps*. IMA Fungus 14(1): 13. 10.1186/s43008-023-00118-5 37415259 PMC10327131

[B101] Zheng P Xia Y-L (2011) Genome sequence of the insect pathogenic fungus *Cordycepsmilitaris*, a valued traditional chinese medicine. Genome Biology 12(11): R166. 10.1186/gb-2011-12-11-r116 PMC3334602 22112802

