# Supplementary material for: ﻿Multi-omics insights into growth and fruiting body development in the entomopathogenic fungus Cordycepsblackwelliae
Source: IMA Fungus. 2025 May 7;16:e147558. doi: 10.3897/imafungus.16.147558 (PMC12079115; doi:10.3897/imafungus.16.147558)
Supplement: Supplementary material 2 — Supplementary figures S1–S16 [file imafungus-16-e147558-s002.docx]

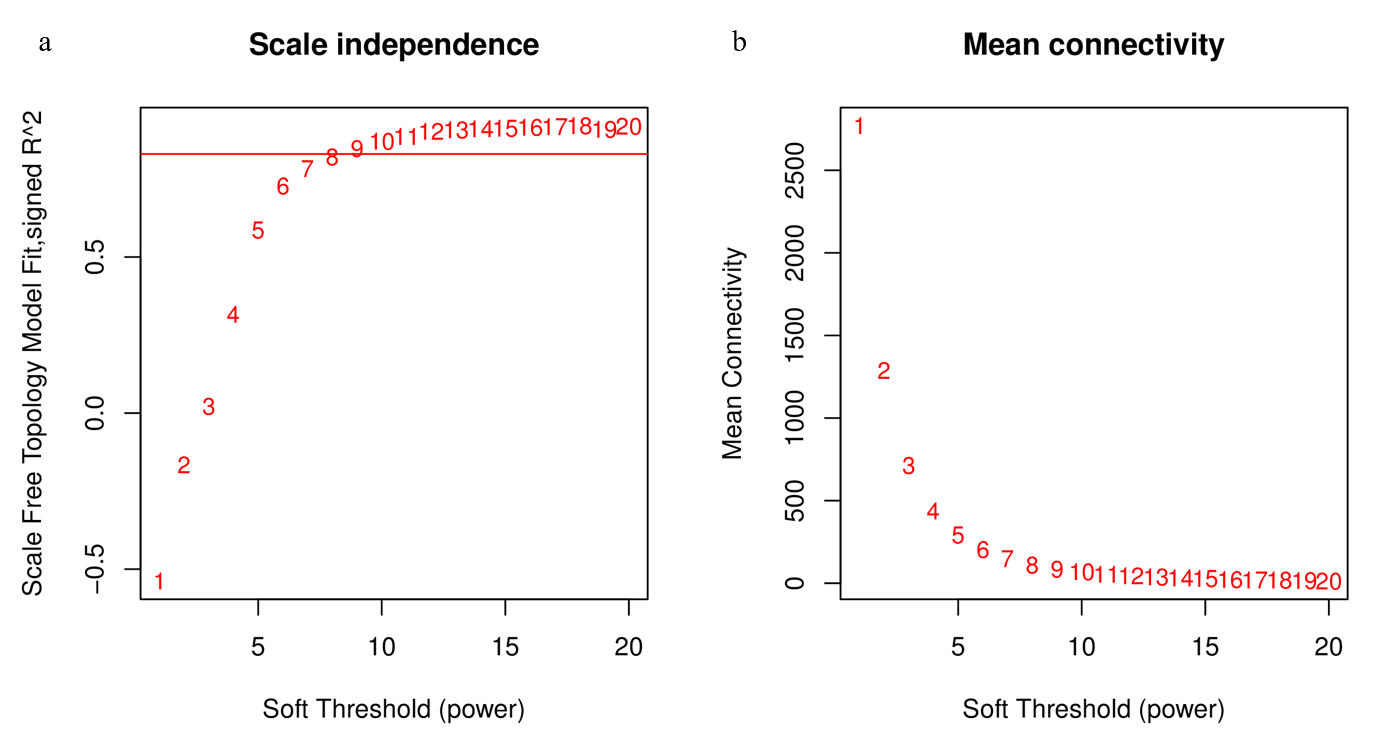


**Fig. S1 Soft threshold determination of WGCNA.** a. The impact of soft threshold power on the scale-free topology model fit index; b. The effect of soft threshold power on the mean connectivity. The optimal soft threshold power was determined to be 8, at which point the network exhibits an asymptotic convergence towards the scale-free network distribution.


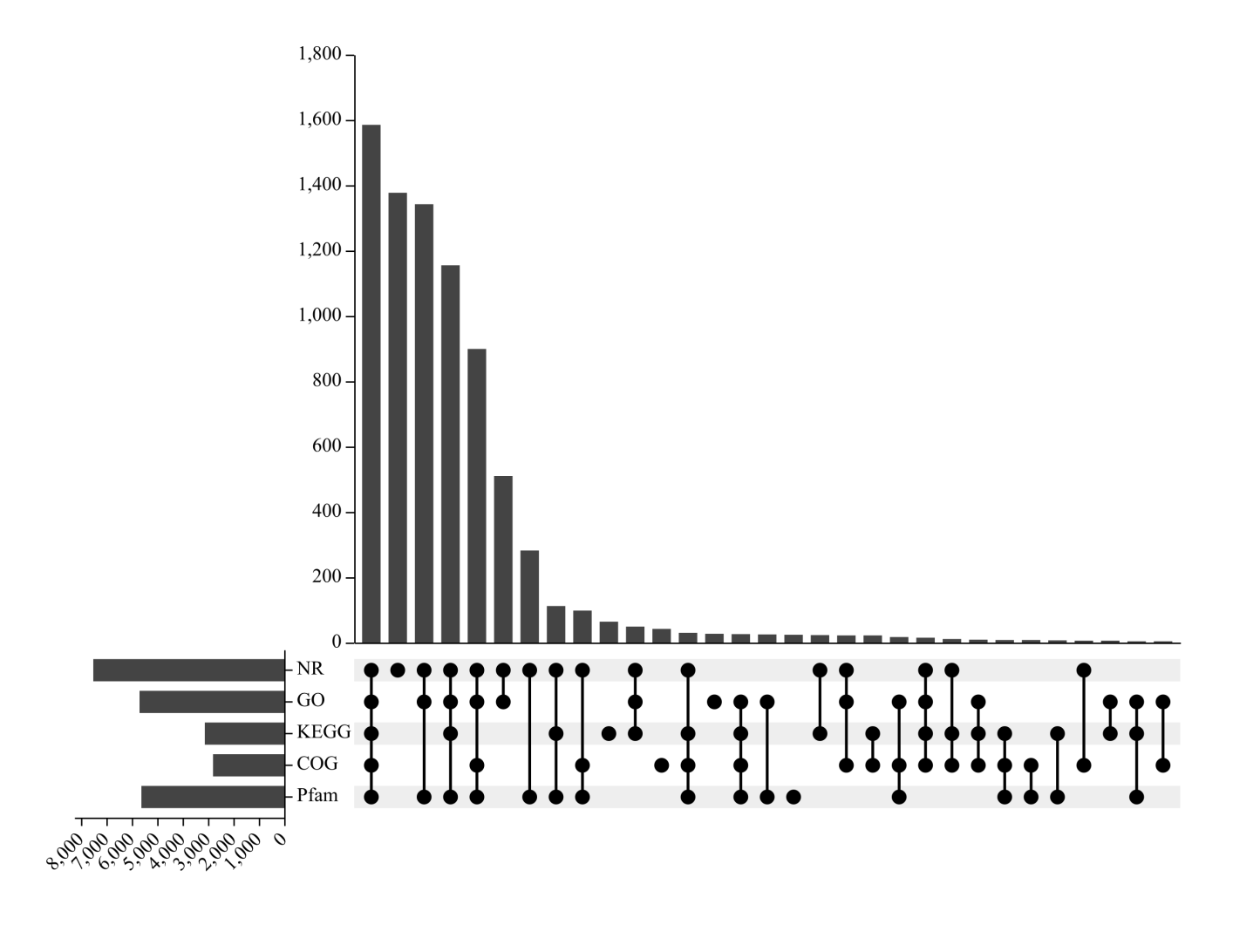


**Fig. S2 Statistics of functional annotations for the *Cordyceps blackwelliae* genome.** The genes identified within the genome were annotated utilizing five distinct databases, namely NR, GO, KEGG, COG, and Pfam. For comprehensive details regarding the annotation summaries, please refer to Supplementary Table S7. In the Upset plot, the horizontal bars indicate the number of annotated genes within each database. A solid dot located in the central matrix signifies the presence of annotated genes linked to a specific database. The intersections of lines illustrate the convergence of multiple databases, with the corresponding values for these intersections represented by vertical bars.


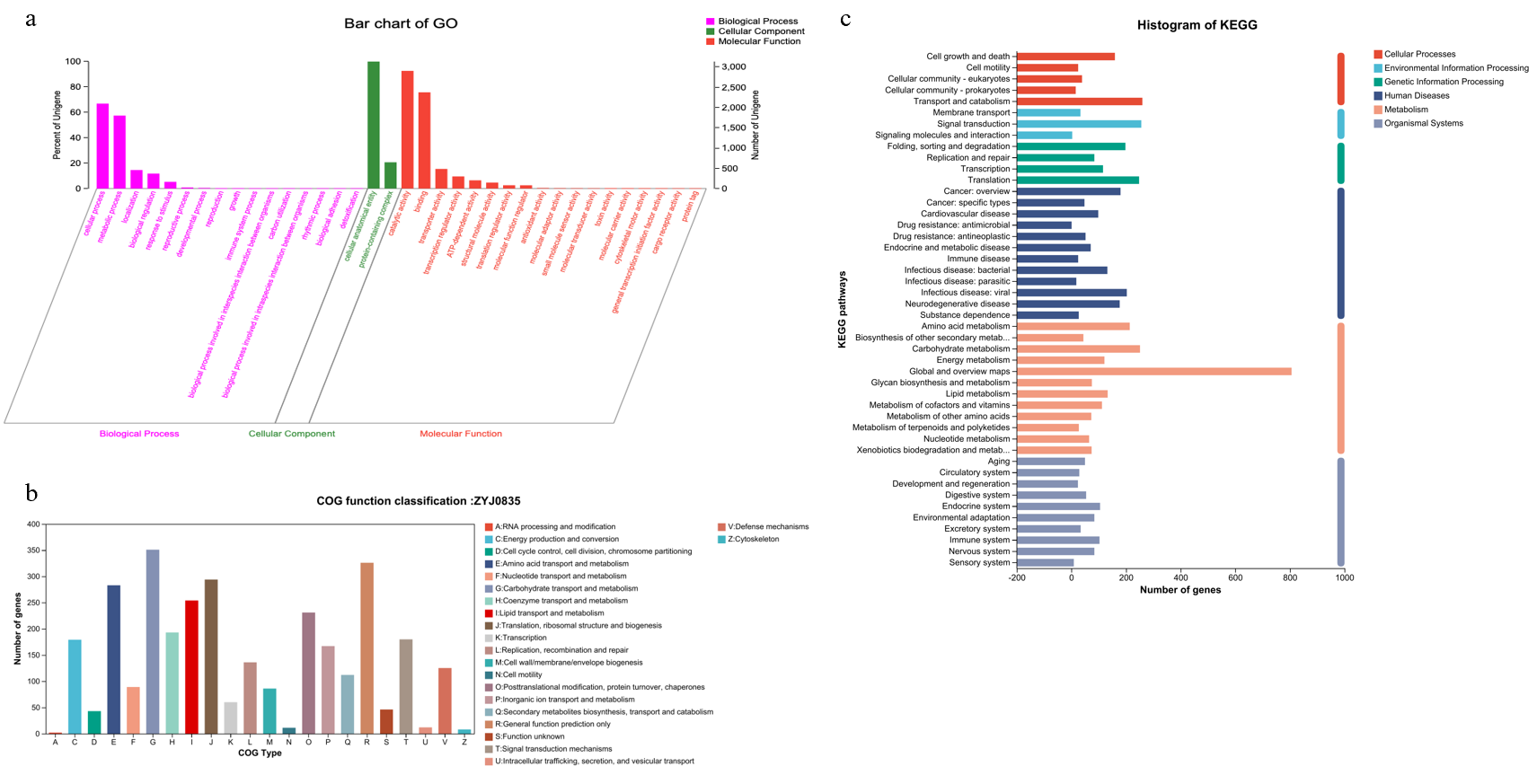


**Fig. S3 Functional annotation of the *Cordyceps blackwelliae* genome.** (a) Gene Ontology (GO). A total of 5,695 protein-coding genes annotated by GO were categorized into 36 subcategories, and divided into three functional categories, i.e., biological process, cellular component, and molecular function. (b) Clusters of Orthologous Groups of proteins (COG). A total of 2,803 protein-encoding genes annotated by COG were categorized into 26 sub-categories (A-Z). (c) Kyoto Encyclopedia of Genes and Genomes (KEGG). A total of 3,126 protein-coding genes annotated by KEGG were divided into six categories, i.e., cellular processes, environmental information processing, genetic information processing, human diseases, metabolism, and organismal systems.

**
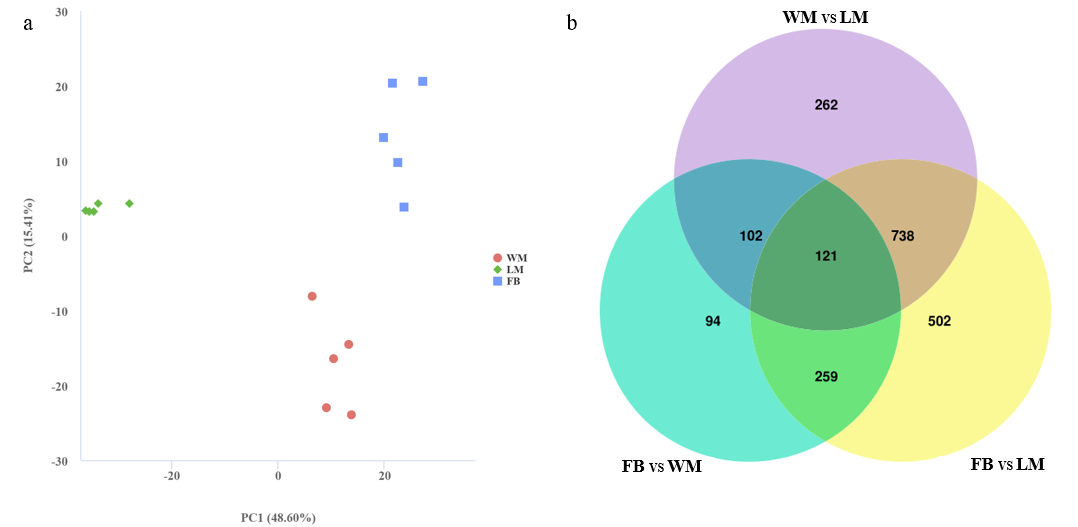
**

**Fig. S4** **Principal component analysis (PCA) and Venn diagram of DEGs in RNA-seq data.** a. PCA for total samples. b. Venn diagram of DEGs by pairwise comparison, |log2FoldChange| > 2.


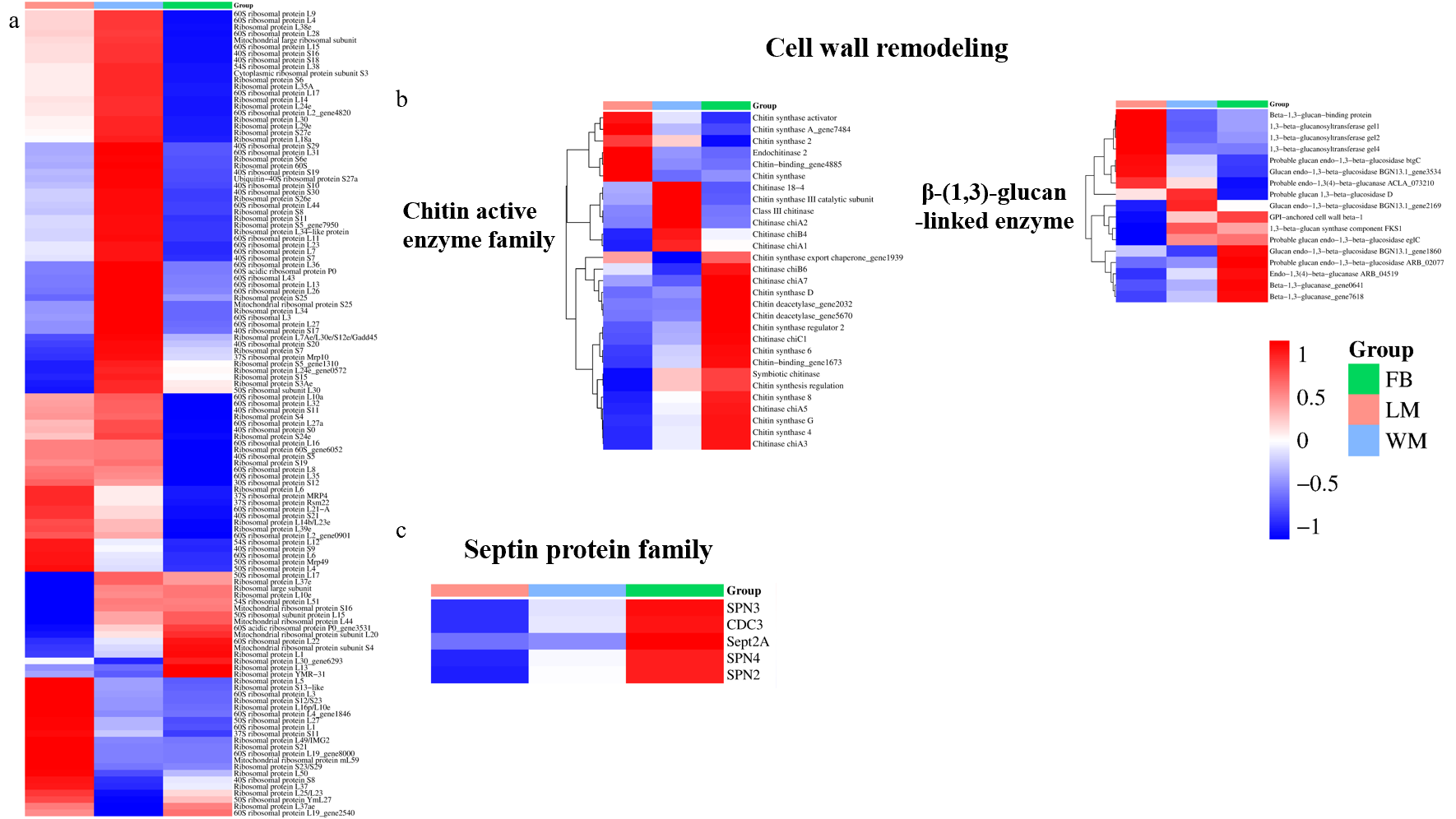


**Fig. S5 Expression heatmap of development-related genes in *Cordyceps blackwelliae*.** (a) Ribosomal genes. (b) Cell wall remodeling related genes. (c) Septin protein family.


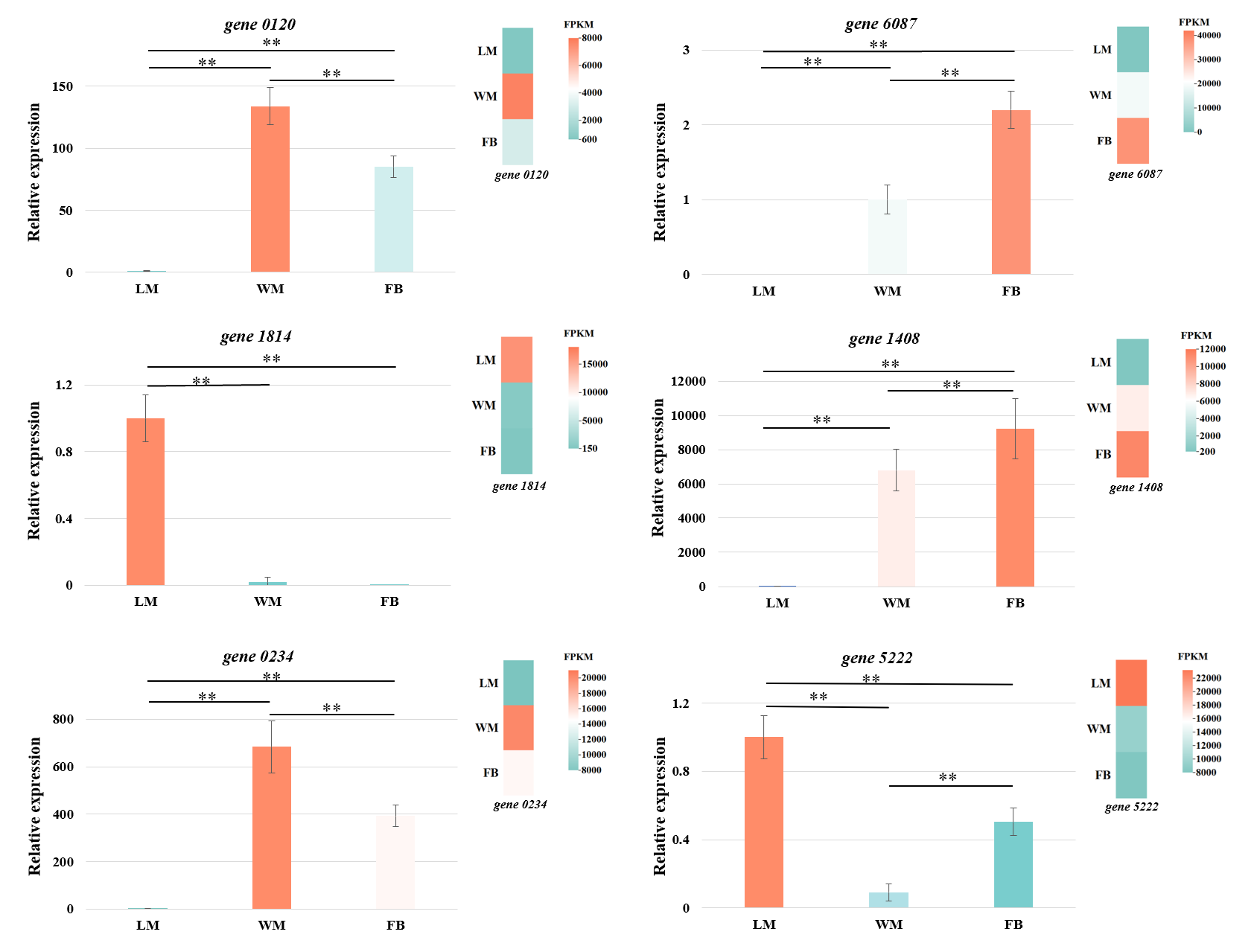


**Fig. S6 Validation of RNA-seq results by RT-qPCR analyses.** The histograms were plotted using data obtained by RT-qPCR. The relative expression of each gene was normalized against the LM stage. Each bar depicts the average value with standard deviations of three independent experiments. **, *P* < 0.01. The FPKM values from RNA-seq analyses are shown in the heat map.


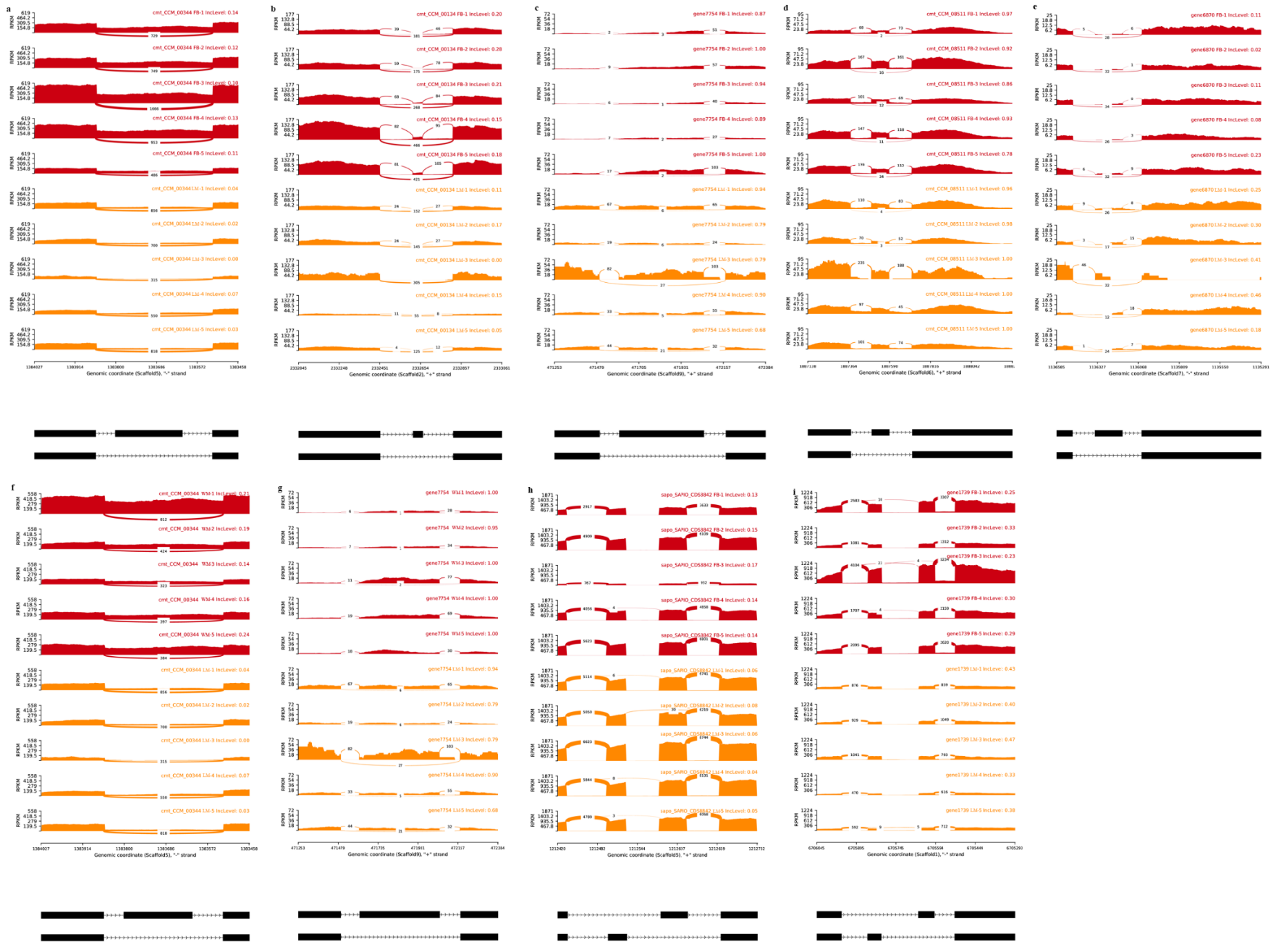


**Fig. S7 Detailed sashimi plots of genes exhibiting developmental stage-specific alternative splicing (FRD < 0.05).** a. CCM_00344, b. CCM_00134, c. gene7754, d.CCM_08511, e. gene6870, f. CCM_00344, g. gene7754, h. SAPIO_CDS8842, i. gene1739. Panels a-e, h, and i represent FB vs LM; panels f and g represent WM vs LM. Panels a-g indicate exon skipping (SE); panels h and I indicate mutually exclusive exon (MXE). Please refer to Supplementary Table S14 for detailed information about alternative splicing.


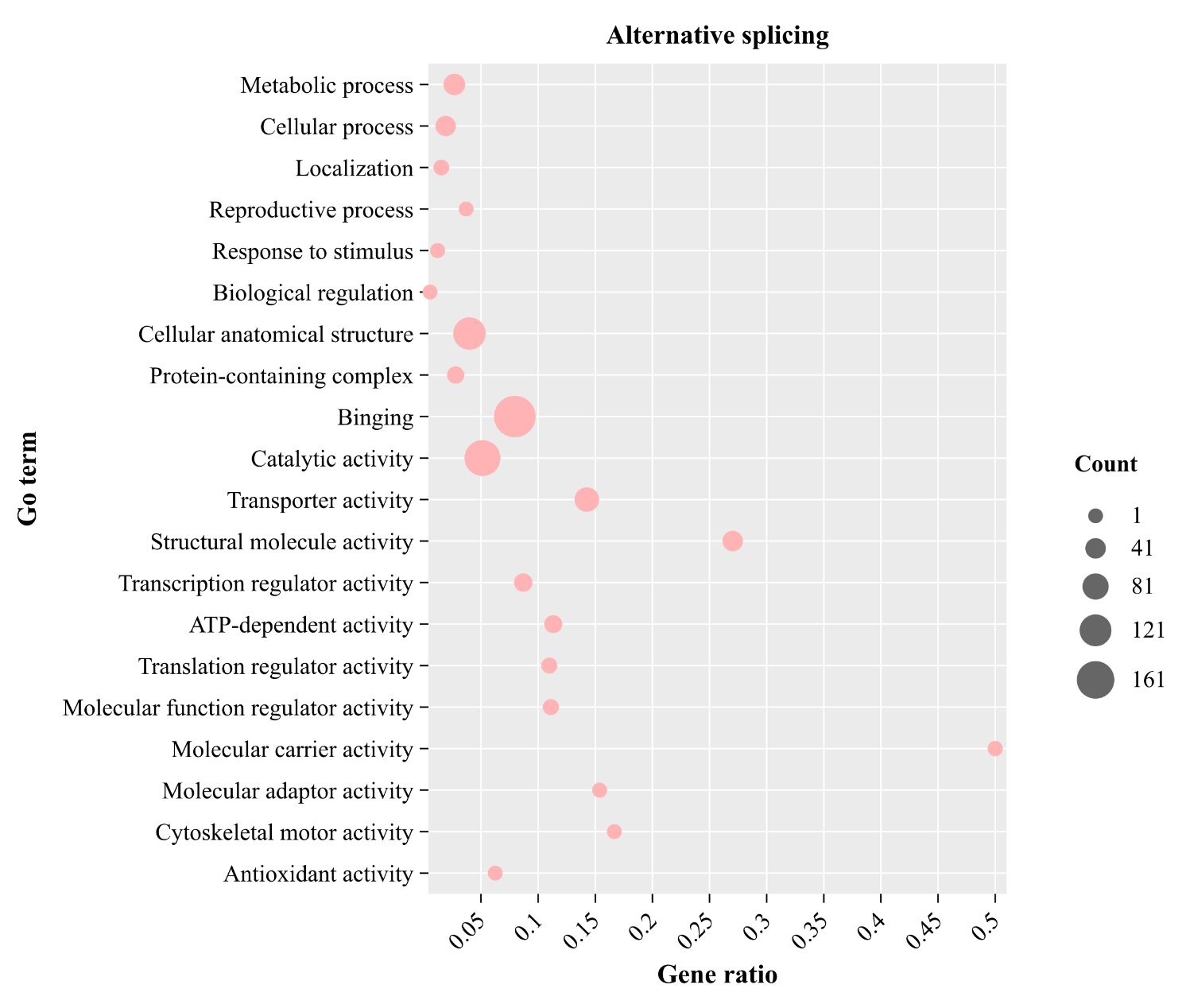


**Fig. S8 GO term enrichment analysis for genes undergoing alternative splicing.** The horizontal axe represents gene ratio, which refers to the proportion of transcripts with alternative splicing relative to all transcripts assigned to a GO term, with higher values indicating more enrichment.

**
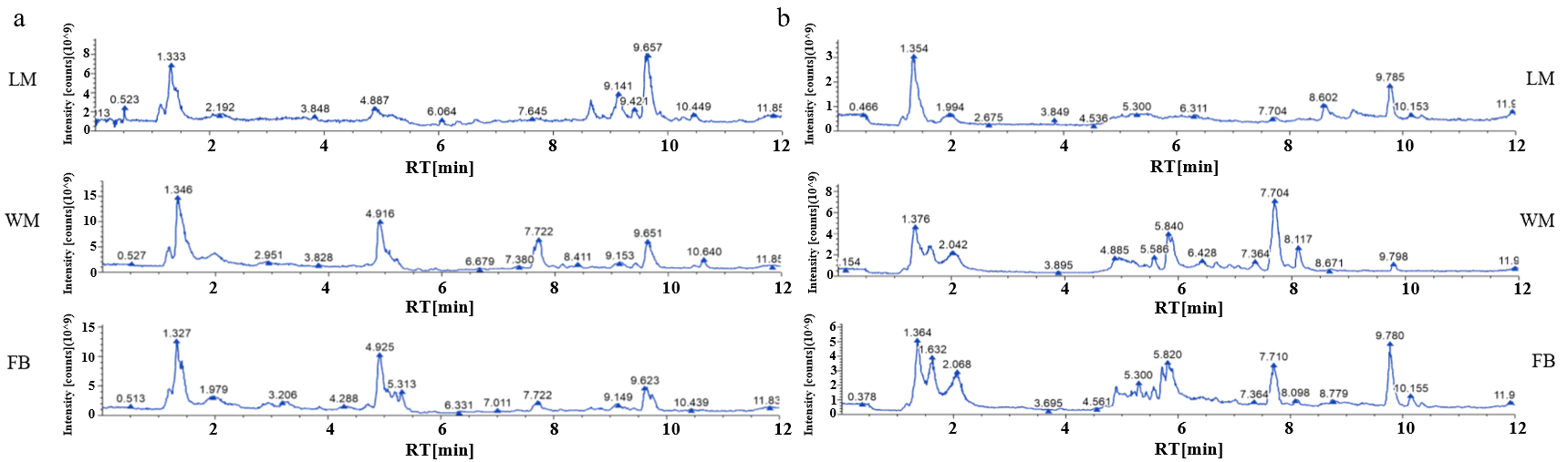
**

**Fig. S9 The total ion chromatogram of three stages in both positive (a) and negative (b) ion modes.**


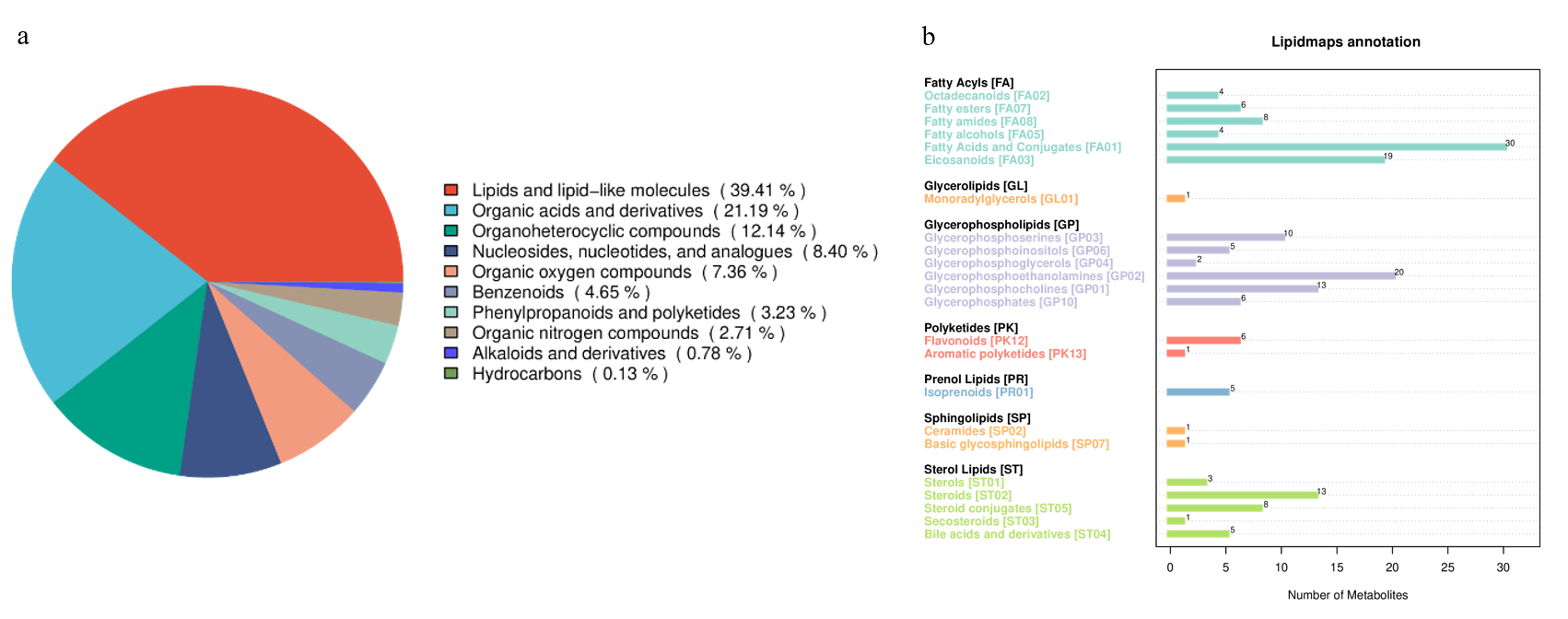


**Fig. S10 Functional annotation of *Cordyceps blackwelliae* metabolites*.*** a. Human Metabolome Database (HMDB). b. LIPID Metabolites and Pathways Strategy (LIPID Maps) database.


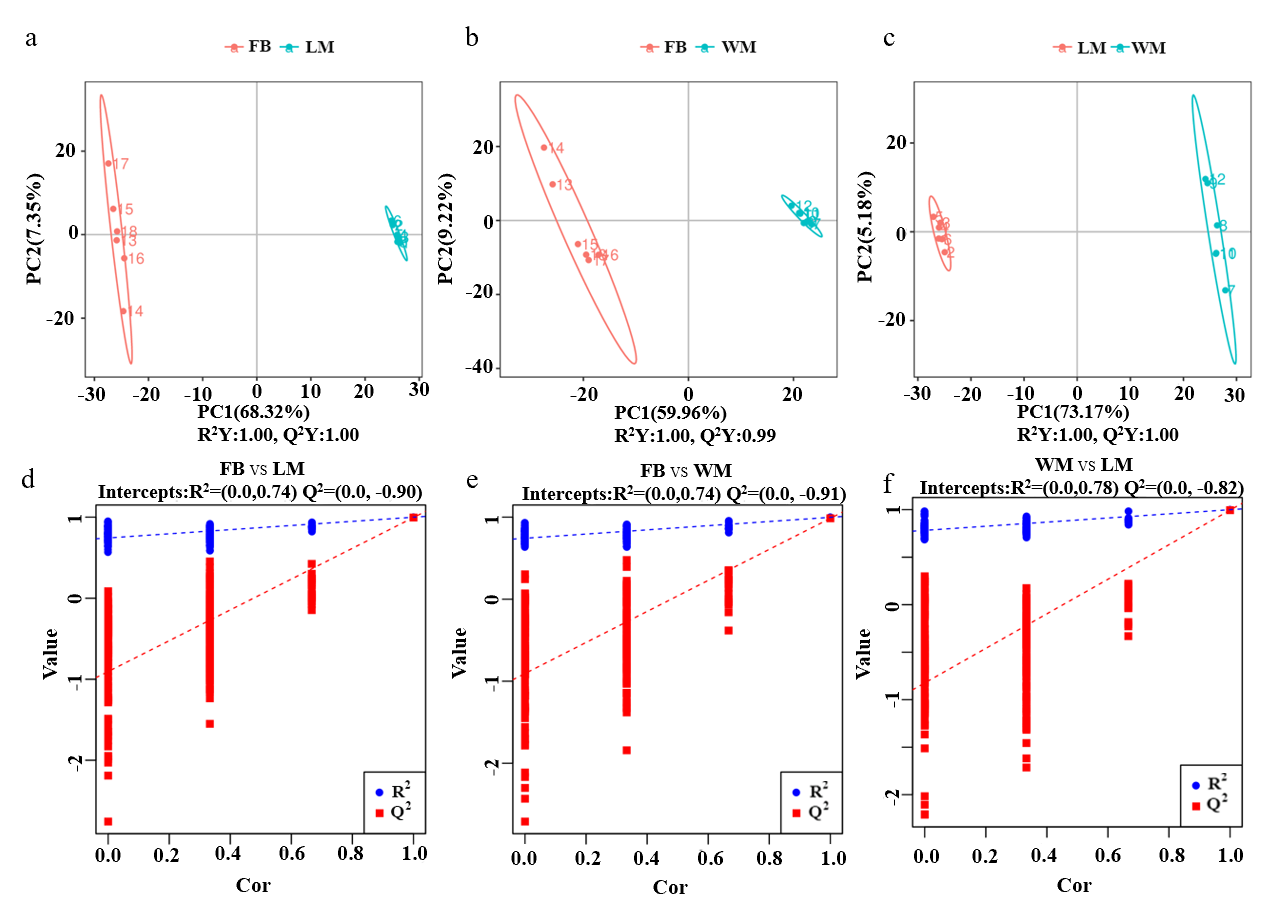


**Fig. S11 Multivariate statistics in three comparisons.** a-c. PLS-DA score plots of samples acquired in positive and negative combined mode. d-f. Validation of the PLS-DA model by permutation testing (200 iterations) in positive and negative combined mode.


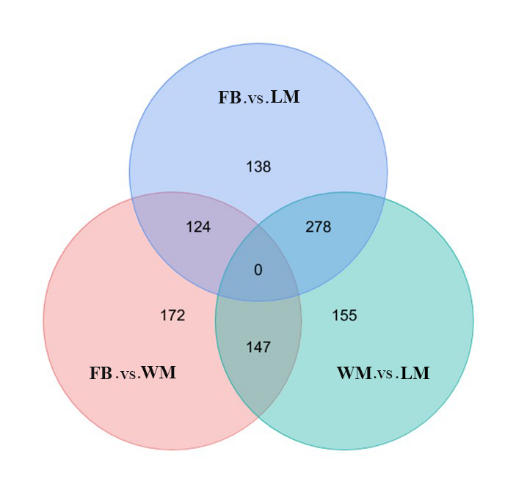


**Fig. S12 Venn diagram of DMs by pairwise comparison.** Variable importance in projection (VIP) > 1, *P*-value < 0.05 and fold change (FC) ≥ 2 or FC ≤ 0.5.


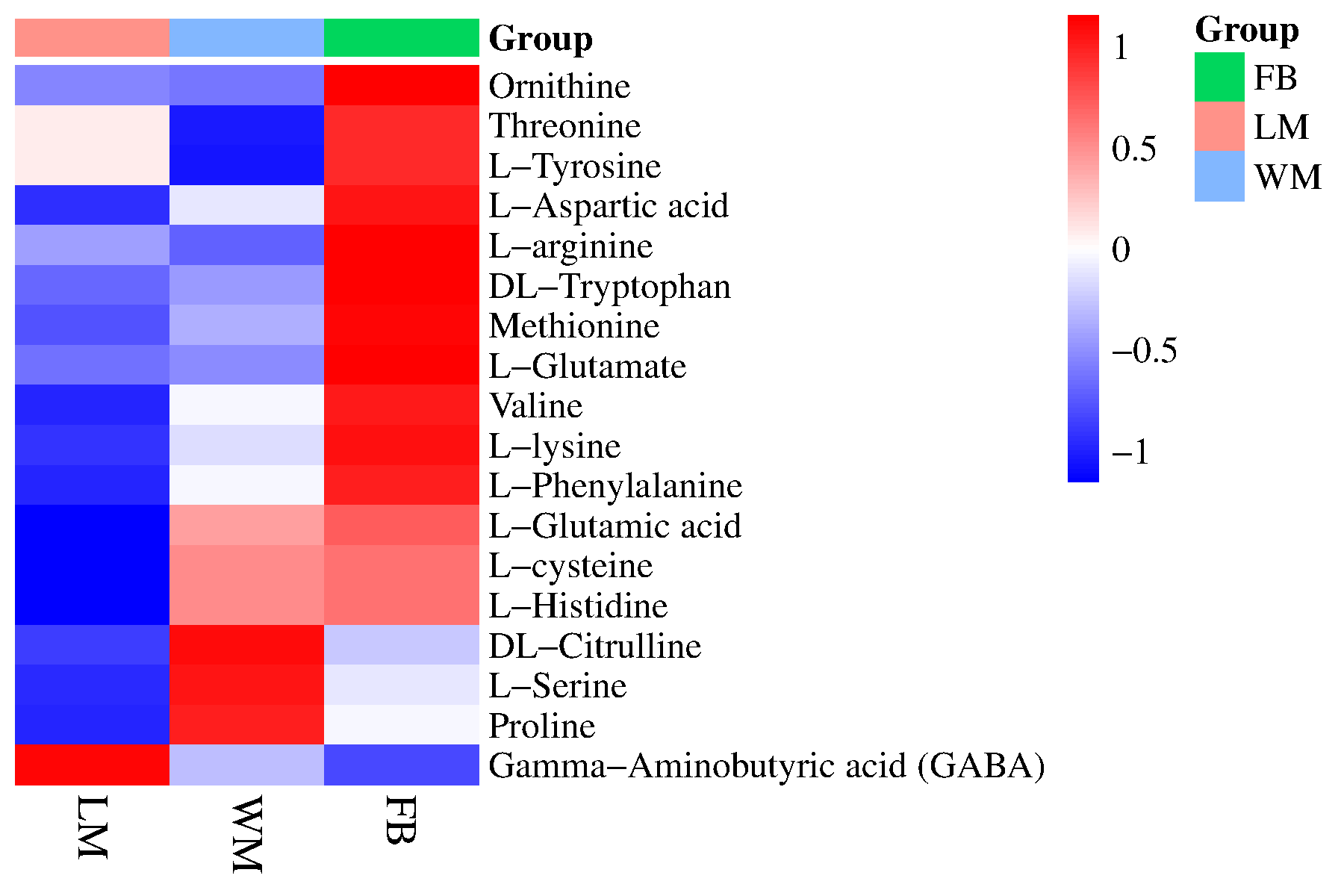


**Fig. S13 Heatmap depicting the differential metabolic profile of amino acids.** Red indicates high metabolite content, and blue represents low content.


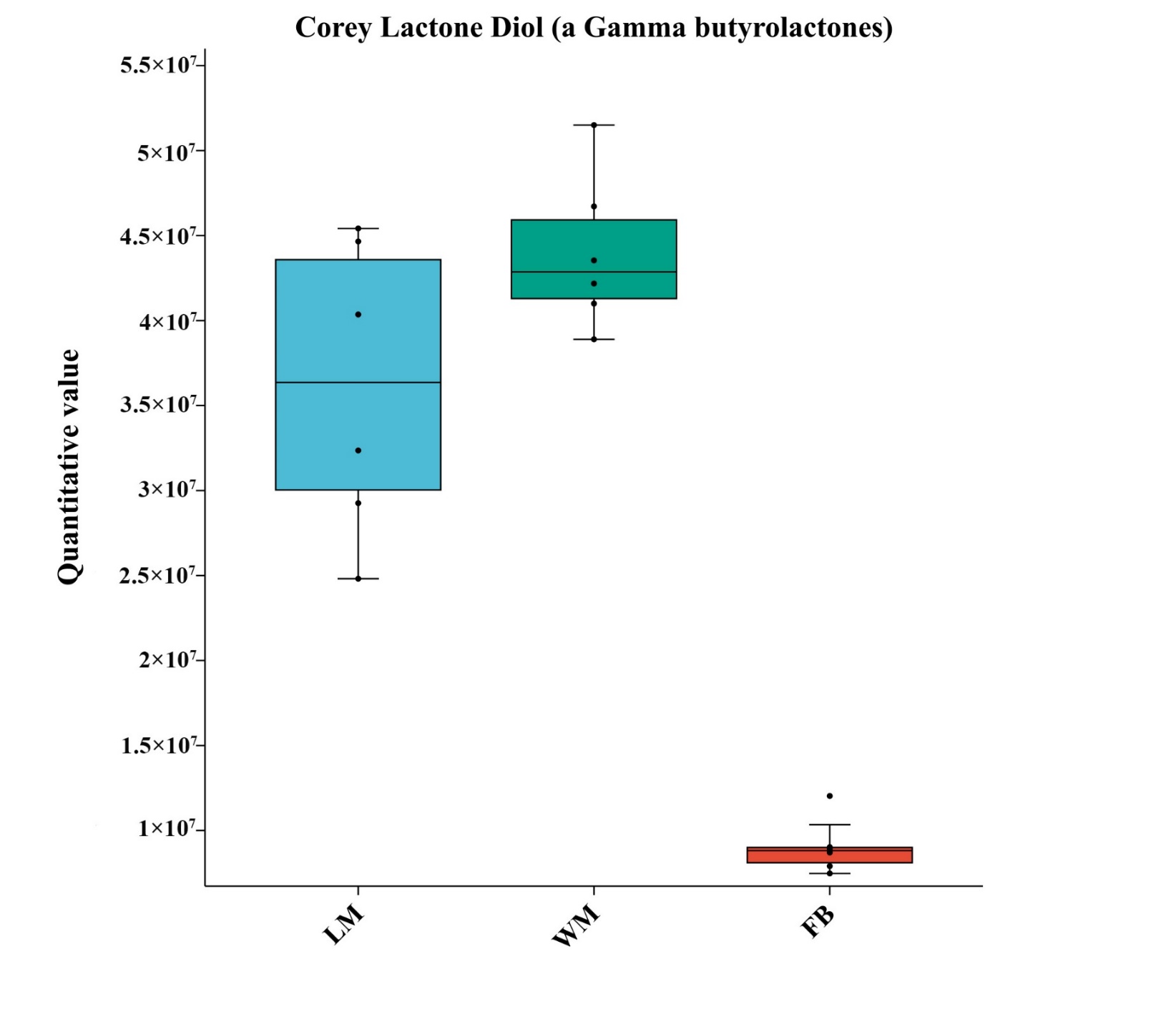


**Fig. S14 Relative content of corey lactone diol across three developmental stages.** The points within the box plot represent the distribution of individual sample measurements.

**
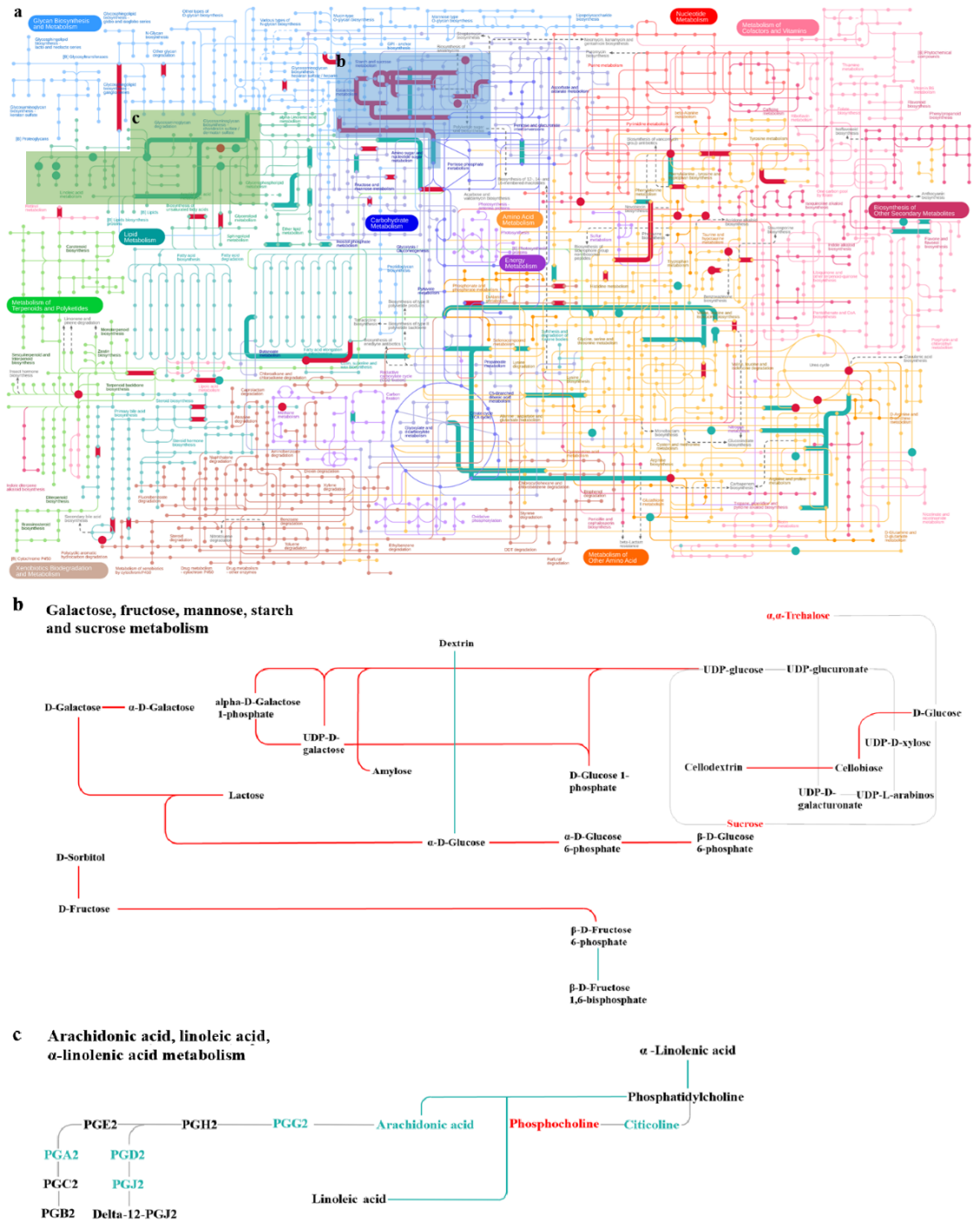
**

**Fig. 15** **Interactive pathway analysis** **of *Cordyceps blackwelliae* in the FB vs. WM comparison.** The bold red lines/dots indicate DEGs/DMs that are up-regulated, while the light seagreen color denotes DEGs/DMs that are down-regulated. The pathways analyzed include: a. Global metabolic pathways; b. Metabolism of galactose, fructose, mannose, starch, and sucrose; c. Metabolism of arachidonic acid, linoleic acid, and α-linolenic acid.


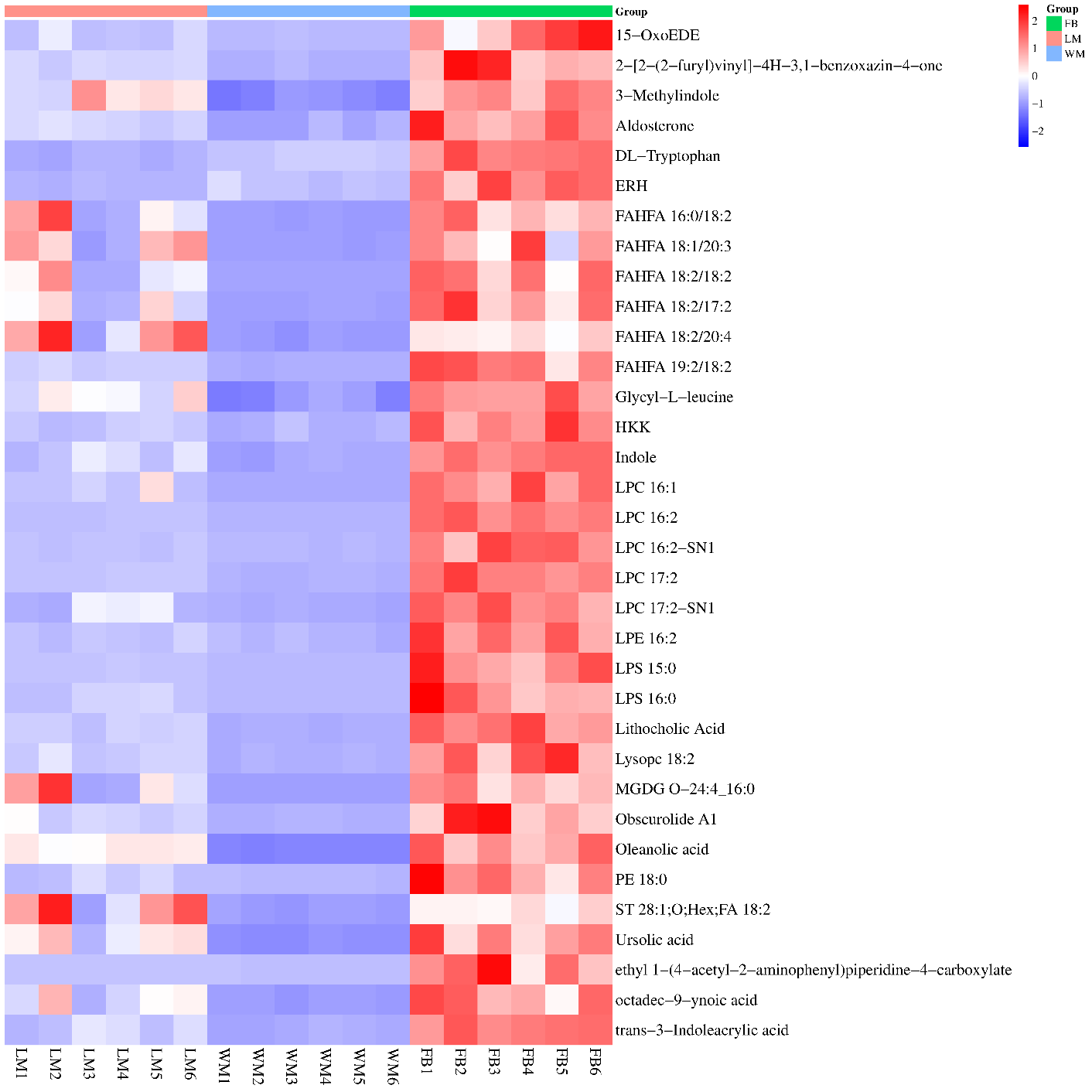


**Fig. S16 Heatmap of 34 metabolites positively correlated with Group1 genes in three stages.**
